# Fuzzy Modeling and Control of HIV Infection

**DOI:** 10.1155/2012/893474

**Published:** 2012-03-22

**Authors:** Hassan Zarei, Ali Vahidian Kamyad, Ali Akbar Heydari

**Affiliations:** ^1^Department of Applied Mathematics, Ferdowsi University of Mashhad, Mashhad 91775-1159, Iran; ^2^Department of Infectious Diseases, Imam Reza Hospital, Mashhad University of Medical Sciences, Mashhad 91379-13316, Iran

## Abstract

The present study proposes a fuzzy mathematical model of HIV infection consisting of a linear fuzzy differential equations (FDEs) system describing the ambiguous immune cells level and the viral load which are due to the intrinsic fuzziness of the immune system's strength in HIV-infected patients. The immune cells in question are considered CD4+ T-cells and cytotoxic T-lymphocytes (CTLs). The dynamic behavior of the immune cells level and the viral load within the three groups of patients with weak, moderate, and strong immune systems are analyzed and compared. Moreover, the approximate explicit solutions of the proposed model are derived using a fitting-based method. In particular, a fuzzy control function indicating the drug dosage is incorporated into the proposed model and a fuzzy optimal control problem (FOCP) minimizing both the viral load and the drug costs is constructed. An optimality condition is achieved as a fuzzy boundary value problem (FBVP). In addition, the optimal fuzzy control function is completely characterized and a numerical solution for the optimality system is computed.

## 1. Introduction

Usage of fuzzy differential equations is a natural way to model dynamical systems under uncertainty [1]. For example these equations are used to modeling the cell growth and dynamic of population [2], dry friction [3], tumor growth [4], and the phenomenon of nuclear disintegration [5] under uncertainty. In [6], transition from HIV to AIDS (the acquired immunodeficiency syndrome) is described through a mathematical model with fuzzy transference rate correlated with the viral load and CD4+ T-cells level by rule bases. Moreover, in [7], the authors have proposed a methodology combining a macroscopic HIV-positive population model, which is a differential equation system whose transference rate from asymptomatic to symptomatic population is found through a fuzzy rule-based system, with an individual microscopic model to study the evolution of positive HIV population for manifestation of AIDS. In [8], a fuzzy delay differential equation is proposed to model HIV infection, assuming that there exists delay between the infection of a CD4+ T-cell by the virus and the production of new virus particles. In this model, the delay and the clearance rate of HIV particles are fuzzy numbers where correlation between them is restated by rule bases. However, it should be noted that the whole parameters of a model such as the production and clearance rate of viruses and immune cells can be the source of uncertainty. In real world, there are various HIV-infected patients with different strengths of immune system causing uncertainty as to the immune cells level and the viral load during the different stages of the disease. A number of mathematical models have been formulated to describe various aspects of the interaction between HIV and the immune cells. The basic and simple model of HIV infection that contains three state variables: healthy CD4+ T-cells, infected CD4+ T-cells, and viruses, is presented by Perelson et al. [9], and more complicated models containing other parts of the immune system such as the cytotoxic T-lymphocyte and the macrophages are presented in [10] and references therein. None of these models can mirror the mentioned uncertainties proposing a mathematical model with fuzzy parameters which could reflect such ambiguities would be desirable.

 One of the earliest suggestions to define the concept of differentiability for fuzzy mappings and, in consequence, to study fuzzy differential equations is the Hukuhara derivative [11]. Nevertheless, the solution of fuzzy differential equation interpreted by Hukuhara derivative became fuzzier as time goes by [12]. Hence, the fuzzy solution behaves quite differently from the crisp solution. In order to overcome this difficulty, a more general definition of derivative for fuzzy-number-valued functions, which is called the strongly generalized differentiability, has been introduced and many papers have been published in this field (see, e.g., [1, 2, 5, 13–17] and references therein). In [13] a generalization of the Hukuhara differentiability to the case of interval-valued functions is introduced and the local existence and uniqueness of solutions for the interval differential equations are obtained under this type of differentiability.

 First-order linear fuzzy differential equations are one of the simplest fuzzy differential equations which may appear in many applications. However, the form of such an equation is very simple, it raises many problems since under different fuzzy differential equation concepts, the behavior of the solutions is different (depending on the interpretation used). This type of equations has been studied by many researchers. For example, the general form of the solutions for the first-order fuzzy differential equations with crisp coefficients under the generalized differentiability concept is presented in [14]. Moreover, an operator method is proposed for solving a class of first- and second-order linear fuzzy differential equations under the assumption of strongly generalized differentiability which is constructed based on their equivalent integral forms [15]. The existence and uniqueness of the solutions has been demonstrated for a first-order linear fuzzy differential equation with impulses subject to boundary value conditions, and the explicit solutions are obtained by calculating the solutions on each level set [16]. The generalized Euler approximation method is applied to solve numerically fuzzy differential equations under generalized differentiability [17]. A number of works in these fields have dealt with the linear fuzzy differential dynamical systems. For instance, in [18], a complex number representation of the **α**-level sets of the linear first-order fuzzy differential dynamical systems where the initial condition is described by a vector of fuzzy numbers is presented and the solutions are obtained under such representation. Using this approach, a method is proposed to find the solutions of a class of linear differential dynamical systems with fuzzy matrices [19] and the proposed method is extended to provide the solutions of linear matrix differential dynamical systems with fuzzy matrices [20].

 Classical control system is described by a differential equation. However, uncertainty is inherit in most dynamic systems. The concept of fuzzy optimal control was presented by Komolov et al. [21] in 1979. Since then, many researchers have studied this type of problems. In [22], fuzzy differential equations are generalized to be fuzzy set control differential equations (FSCDEs) and the problem of stability and controllability of FSCDE are presented. Furthermore, some properties of the fuzzy solution for the linear FSCDE as well as the necessary and sufficient optimality conditions for a linear fuzzy time optimal control problem are obtained in [23].

 In this paper, we model the uncertain behaviors of CD4+ T-cells and CTLs level and the viral load in different patients by a system of linear fuzzy differential equations and analyze the optimal control regarding minimizing both the viral load and drug costs.

 Following a preliminary introduction, in Section 3, a system of linear differential equations with fuzzy parameters describing the ambiguous behaviors of CD4+ T- cells and CTLs level and the HIV viral load in patients with a weak, moderate and strong immune system is introduced. Moreover, a method for finding explicit solutions to the proposed model is introduced in this section. Some authors have used mathematical models for HIV infection in conjunction with control theory to achieve appropriate goals. Although the proposed model is simple, it can be used to investigate the effects of antiretroviral therapy in preventing the HIV progression.  Section 4 is devoted to the latter topic. The last section deals with the conclusion.

## 2. Preliminaries

In this section, we give some definitions and introduce the necessary notations which will be used throughout the paper. See, for example, [12].

**Definition 1**

(fuzzy set, **α**-level set, and fuzzy number) A fuzzy set $\tilde{u}$ in *R* ^*n*^ is defined as a set of all pairs $\left(x,\mu_{\tilde{u}\;}(x))\in R^{n}\times [0,\;1\right]$ for some function $\mu_{\tilde{u}}:R^{n}\to [0,\;1]$, which is called the membership function of $\tilde{u}$, and $\mu_{\tilde{u}}(x)$ is interpreted as the membership grade of a element *x* in the fuzzy set $\tilde{u}$. We define ${\left[\tilde{u}\right]}^{\alpha }=\{x\in R|\mu_{\tilde{u}}(x)\geq \alpha \}$ the **α**-level set of $\tilde{u}$, with 0 < *α* ≤ 1. For *α* = 0, the support of $\tilde{u}$ is defined as ${\left[\tilde{u}\right]}^{0}=\mathrm{supp}(\tilde{u})=\mathrm{cl}\{\;x\in R|\mu_{\tilde{u}}(x)>0\}$, where cl denotes the closure of a subset. A fuzzy set $\tilde{u}$ in *R* is called a fuzzy number in *R* if

- $\mu_{\tilde{u}}$ is upper semicontinuous on *R*,
- $\tilde{u}$ is a convex fuzzy set, that is, $\mu_{\tilde{u}}\left(\lambda x+\left(1-\lambda \right)y\right)\geq \min\left\{\mu_{\tilde{u}}\left(x\right),\mu_{\tilde{u}}\left(y\right)\right\}$, for all *x*, *y* ∈ *R*, *λ* ∈ [0, 1],
- $\mu_{\tilde{u}}$ is normal, that is, there exists a unique *x*_0_ ∈ *R* such that $\mu_{\tilde{u}}(x_{0})=1$,
- the support of $\tilde{u}$ is compact.

**Example 2**

**Figure 1:**
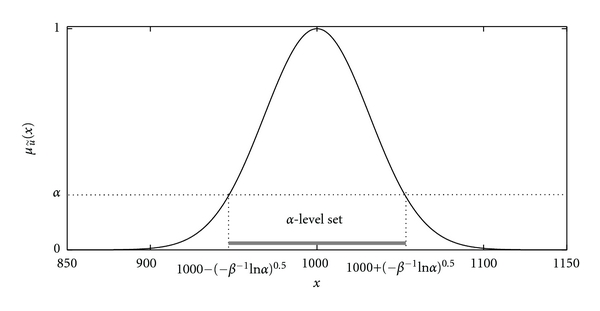
The membership function of the fuzzy set of real numbers “close to 1000” and its **α**-level set.

The normal level of CD4+ T-cells in blood, that is: “close to 1000 cells/**μ**L,” can be represented as a fuzzy set $\tilde{u}$ with a membership function defined as $\mu_{\tilde{u}}(x)=\exp(-\beta {\left(x-1000\right)}^{2})$ where  **β** is a positive real number. It is easy to see that ${\left[\tilde{u}\right]}^{\alpha }=[1000-\sqrt{-\beta^{-1}\ln\alpha },\;1000+\sqrt{-\beta^{-1}\ln\alpha }]$. The membership function $\mu_{\tilde{u}}(\cdot )$ with *β* = 0.0005 and ${\left[\tilde{u}\right]}^{\alpha }$ are shown in Figure 1. Note that ${\left[\tilde{u}\right]}^{0}$ = (−*∞*, +*∞*); hence, $\tilde{u}$ is not a fuzzy number.

**Figure 2:**
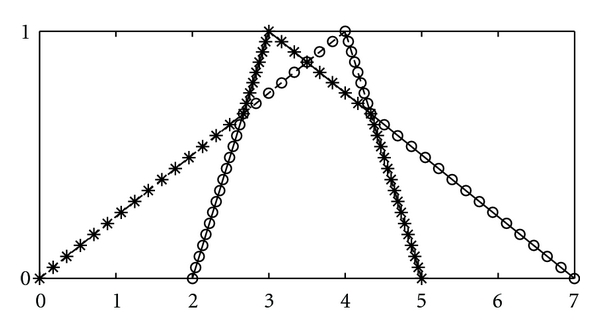
The fuzzy numbers $\tilde{u}=(4,4,1)$ (- - -), $\tilde{v}=(3,1,4)$ (—), $\tilde{\max}\{\tilde{u},\tilde{v}\}$ (∘∘), and $\tilde{\min}\{\tilde{u},\tilde{v}\}$ (∗∗).

**Figure 3:**
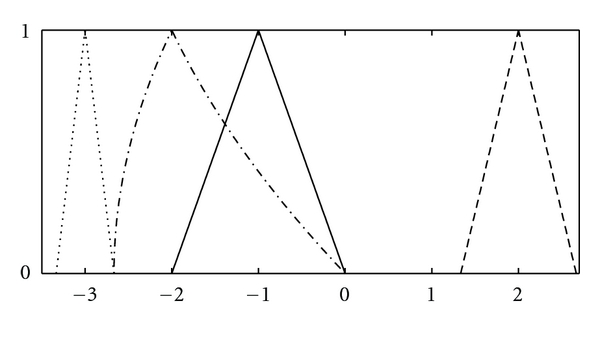
The fuzzy numbers $\tilde{u}=(-1,1)$ (—), $\tilde{v}=(2,2/3)$ (- - -) , $\tilde{u}\Theta_{H}\tilde{v}$ (*⋯*), and $\tilde{u}{⊗}_{Z}\tilde{v}$ (–·–).

The set of all fuzzy numbers in *R* is denoted by *F*(*R*). From (i)–(iv), it follows that if $\tilde{u}$ belongs to *F*(*R*) then the **α** -level set ${\left[\tilde{u}\right]}^{\alpha }$ is a nonempty compact interval for all *α* ∈ [0, 1]. The notation ${\left[\tilde{u}\right]}^{\alpha }=[{\underline{u}}_{\alpha },{\overset{̅}{u}}_{\alpha }]$ denotes explicitly the **α**-level set of $\tilde{u}$. Triangular fuzzy numbers are one of the most commonly used fuzzy numbers. The membership function of a triangular fuzzy number $\tilde{u}$ is completely characterized with the peak (or center)* m*, left width *σ* ≥ 0, and right width *β* ≥ 0 and has the following form:

(1) $$\mu_{\tilde{u}}\left(x\right)=\{\begin{array}{cc} 1-\frac{m-x}{\sigma }, & \text{if}\;m-\sigma \leq x<m,\\ 1-\frac{x-m}{\beta }, & \text{if}\;m\leq x\leq m+\beta ,\\ 0, & \text{otherwise}, \end{array}$$

and we use the notation $\tilde{u}=(m,\sigma ,\beta )$. A triangular fuzzy number $\tilde{u}=(m,\sigma ,\beta )$ is named a symmetric triangular fuzzy number if its left width and right width are equal and we denote it by $\tilde{u}=(m,\sigma )$, for brevity. Some examples of triangular and symmetric triangular fuzzy numbers are depicted in Figures 2 and 3, respectively. We consider a crisp number *a* ∈ *R* as a symmetric triangular fuzzy number *a* = (*a*, 0).

Let *f* be a real-valued mapping on *R* ^*n*^. Assume ${\tilde{u}}_{j}$, *j* = 1,…, *n* are fuzzy numbers in *R*. Using the extension principle, we can define $\tilde{Y}=f({\tilde{u}}_{1},\ldots ,{\tilde{u}}_{n})$ as a fuzzy set in *R* such that

(2) $$\begin{array}{cc} & \mu_{\tilde{Y}}\left(y\right)\\ & =\{\begin{array}{cc} \sup\;\min\{\mu_{{\tilde{u}}_{1}}\left(x_{1}\right),\ldots ,\mu_{{\tilde{u}}_{n}}\left(x_{n}\right)\;|\; & \\ \;\;\;\;\left(x_{1},\ldots ,x_{n}\right)\in f^{-1}\left(y\right)\}, & \text{if}\;f^{-1}\left(y\right)\neq \phi ,\\ 0, & \;\text{if}\;f^{-1}\left(y\right)=\phi . \end{array} \end{array}$$

**Example 3**

Let *f*(*x*) = *λx* be a linear function. Suppose $\tilde{u}\in F(R)$, and let $\tilde{Y}=f(\tilde{u})$. Then, using the extension principle, we obtain $\mu_{\tilde{Y}}(y)=\sup\{\mu_{\tilde{u}}(x)|y=\lambda x\}=\mu_{\tilde{u}}(y/\lambda )$. Especially, if *λ* = −1, then we write $f(\tilde{u})=\Theta \tilde{u}$ and we have $\mu_{\Theta \tilde{u}}(y)=\mu_{\tilde{u}}(-y)$.

**Theorem 4**

Let *f* be a real-valued mapping on *R* ^*n*^, and let ${\tilde{u}}_{j}$, *j* = 1,…, *n*, be fuzzy numbers in *R*. Then, ${\left[f({\tilde{u}}_{1},\ldots ,{\tilde{u}}_{n})\right]}^{\alpha }=f({\left[{\tilde{u}}_{1}\right]}^{\alpha },\ldots ,{\left[{\tilde{u}}_{n}\right]}^{\alpha })$ where $f({\tilde{u}}_{1},\ldots ,{\tilde{u}}_{n})$ is defined by the extension principle and $f({\left[{\tilde{u}}_{1}\right]}^{\alpha },\ldots ,{\left[{\tilde{u}}_{n}\right]}^{\alpha })=\{f(u_{1},\ldots ,u_{n})|u_{\;1}\in {\left[{\tilde{u}}_{1}\right]}^{\;\alpha },\ldots ,u_{n}\in {\left[{\tilde{u}}_{n}\right]}^{\;\alpha }\}$.

**Proof**

The above theorem defines arithmetic operations of fuzzy numbers in terms of their **α**-level sets by ${\left[\tilde{u}⊕\tilde{v}\right]}^{\alpha }=[{\underline{u}}_{\alpha }+{\underline{v}}_{\alpha },{\overset{̅}{u}}_{\alpha }+{\overset{̅}{v}}_{\alpha }]$, ${\left[\tilde{u}\;\Theta \tilde{\;v}\right]}^{\alpha }=[{\underline{u}}_{\alpha }-{\overset{̅}{v}}_{\alpha },{\overset{̅}{u}}_{\alpha }-{\underline{v}}_{\alpha }]$, and ${\left[\tilde{u}⊗\tilde{v}\right]}^{\alpha }=$ $\left[\min\{{\underline{u}}_{\alpha }{\underline{v}}_{\alpha },{\underline{u}}_{\alpha }{\overset{̅}{v}}_{\alpha },{\overset{̅}{u}}_{\alpha }{\underline{v}}_{\alpha },{\overset{̅}{u}}_{\alpha }{\overset{̅}{v}}_{\alpha }\right\}$, $\max\{{\underline{u}}_{\alpha }{\underline{v}}_{\alpha },{\underline{u}}_{\alpha }{\overset{̅}{v}}_{\alpha },{\overset{̅}{u}}_{\alpha }{\underline{v}}_{\alpha },{\overset{̅}{u}}_{\alpha }{\overset{̅}{v}}_{\alpha }\}]$, where ⊕, Θ, and ⊗ denote the addition, minus, and multiplication operators on *F*(*R*), respectively. Moreover, ${\left[\Theta \;\tilde{u}\right]}^{\alpha }=[-{\overset{̅}{u}}_{\alpha },-{\underline{u}}_{\alpha }]$.

**Definition 5 (fuzzy max, fuzzy min, fuzzy inequality, and the weighted center of gravity)**

Let $\tilde{u},\tilde{v}\in F(R)$, and *f*(*x*, *y*) = max⁡{*x*, *y*}. Then, the maximum of $\tilde{u}$ and $\tilde{v}$ is defined by $f(\tilde{u},\tilde{v})$ and applying the extension principle. Similarly, setting *f*(*x*, *y*) = min⁡{*x*, *y*}, then $f\;(\tilde{u},\tilde{v})$ defines the minimum of $\tilde{u}$ and $\tilde{v}$. We denote the maximum and the minimum of $\tilde{u}$ and $\tilde{v}$ by $\tilde{\max}\{\tilde{u},\tilde{v}\}$ and $\tilde{\min}\{\tilde{u},\tilde{v}\}$, respectively. The notation “$\tilde{\leq }$” will be used for the inequality relation between fuzzy numbers $\tilde{u}$ and $\tilde{v}$ and is defined as $\tilde{u}\;\tilde{\leq }\;\tilde{v}\Leftrightarrow$ $\text{m}\tilde{\text{i}}\text{n}\{\tilde{u},\tilde{v}\}=\tilde{u}$. Moreover, the quantity of fuzzy number $\tilde{u}$ can be given by its weighted center of gravity (WCOG) defined as $u=\int_{0}^{1}\alpha ({\overset{̅}{u}}_{\alpha }+{\underline{u}}_{\alpha })d\alpha$, where the weights are the membership degrees.

**Example 6**

Two triangular fuzzy numbers $\tilde{u}=(4,4,1)$, $\tilde{v}=(3,1,4)$ and their maximum and minimum are shown in Figure 2. Moreover, if $\tilde{u}=(m,\sigma ,\beta )$, then the WCOG of $\tilde{u}$ is *u* = ∫_0_^1^ *α*[2 *m* + (1 − *α*)(*σ* − *β*)]*dα* = *m* + 1/6(*σ* − *β*).

Applying Theorem 4, we get ${\left[\text{m}\tilde{\text{i}}\text{n}(\tilde{u},\tilde{v})\right]}^{\alpha }=\min\{{\left[\tilde{u}\right]}^{\alpha },{\left[\tilde{v}\right]}^{\alpha }\}=[\min\{{\underline{u}}_{\alpha },{\underline{v}}_{\alpha }\},\;\min\{{\overset{̅}{u}}_{\alpha },{\overset{̅}{v}}_{\alpha }\}]$; hence, $\tilde{u}\tilde{\leq }\tilde{v}\Leftrightarrow {\overset{̅}{u}}_{\alpha }\leq {\overset{̅}{v}}_{\alpha }$, ${\underline{u}}_{\alpha }\leq {\underline{v}}_{\alpha }$, for all *α* ∈ [0, 1].

**Definition 7 (H-difference and Z-product)**

Let $\tilde{u},\tilde{v}\in F(R)$. If there exists $\tilde{z}\in F(R)$ such that $\tilde{u}=\tilde{v}\;⊕\;\tilde{z}$, then $\tilde{z}$ is called the H-difference of $\tilde{u}$ and $\tilde{v}$ and it is denoted by $\tilde{u\;}\Theta_{H}\;\tilde{v}$. Moreover, if there exist $\tilde{z}\in F(R)$ such that $\tilde{z}⊗{\tilde{v}}^{\;-1}=\tilde{u}$, then we call it the Z-product of $\tilde{u}$ and $\tilde{v}$ and we denote it by $\tilde{z}=\tilde{u}\;{⊗}_{Z}\;\tilde{v}$.

Note that the Z-product of fuzzy numbers is a new concept which is introduced in this paper for the first time. It is easy to see that ${\left[\tilde{u}\;\Theta_{H}\;\tilde{v}\right]}^{\alpha }=[{\underline{u}}_{\alpha }-{\underline{v}}_{\alpha },{\overset{̅}{u}}_{\alpha }-{\overset{̅}{v}}_{\alpha }]$ and ${\left[\tilde{u}\;{⊗}_{Z}\;\tilde{v}\right]}^{\alpha }=[{\underline{u}}_{\alpha }{\underline{v}}_{\alpha },{\overset{̅}{u}}_{\alpha }{\overset{̅}{v}}_{\alpha }]$, if $\tilde{u}\;\tilde{\leq }\;(0,0)$ and $\left(0,0\right)\;\tilde{\leq }\;\tilde{\;v}$.

**Example 8**

Let $\tilde{u}=(-1,1)$ and $\tilde{v}=(2,2/3)$. A straightforward calculation shows ${\left[\tilde{u\;}\Theta_{H}\;\tilde{v}\right]}^{\alpha }=[-3-1/3(1-\alpha ),-3+1/3(1-\alpha )]$; hence, $\tilde{u}\;\Theta_{H}\;\tilde{v}=(-3,1/3)$. Furthermore, we obtain ${\left[\tilde{u}{⊗}_{Z}\tilde{v}\right]}^{\alpha }=[-8/3+2/3\alpha^{2},-8/3\alpha +2/3\alpha^{2}]$ which is valid **α**-level set of a fuzzy number (not a triangular fuzzy number). The fuzzy numbers $\tilde{u}$, $\tilde{v}$ and their H-difference and Z-product are shown in Figure 3, for the sake of clarity.

**Definition 9 (the strongly generalized differentiability)**

Let $\tilde{x}:(a,b)\to F(R)$ be a fuzzy function and *t*_0_ ∈ (*a*, *b*). We say that. $\tilde{x}$ is differentiable at *t*_0_ if it exists an element $\dot{\tilde{x}}\left(t_{0}\right)\in F\left(R\right)$ such that, for all *h* > 0 sufficiently near to 0,

- there are $\tilde{x}\left(t_{0}+h\right)\Theta_{H}\;\tilde{x}(t_{0})$ and $\tilde{x}\left(t_{0}\right)\Theta_{H}\;\tilde{x}(t_{0}-h)$ and the limits $\lim_{h\to 0^{+}}\left(\tilde{x}\left(t_{0}+h\right)\Theta_{H}\;\tilde{x}(t_{0})/h\right)=\lim_{h\to 0^{+}}\left(\tilde{x}\left(t_{0}\right)\Theta_{H}\;\tilde{x}(t_{0}-h)/h\right)=\dot{\tilde{x}}\left(t_{0}\right)$ or
- there are $\tilde{x}\left(t_{0}-h\right)\Theta_{H}\;\tilde{x}(t_{0})$ and $\tilde{x}\left(t_{0}\right)\Theta_{H}\;\tilde{x}(t_{0}+h)$ and the limits $\text{l}{\text{im}}_{h\to 0^{+}}\left(\tilde{x}(t_{0})\Theta_{H}\tilde{x}(t_{0}+h)/-h\right)=\lim_{h\to 0^{+}}\left(\tilde{x}(t_{0}-h)\Theta_{H}\tilde{x}(t_{0})/-h\right)=\dot{\tilde{x}}\left(t_{0}\right)$, where the limits are taken in the metric *D* defined as $D\left(\tilde{u},\tilde{v}\right)=\sup_{\alpha \in [0,1]}\max\left\{\left|{\overset{̅}{u}}_{\alpha }-{\overset{̅}{v}}_{\alpha }\right|,\left|{\underline{u}}_{\alpha }-{\underline{v}}_{\alpha }\right|\right\}$, for all $\tilde{u},\tilde{v}\in F(R)$.

**Theorem 10**

If $\tilde{x}(t)$ is differentiable in the first form (i), then ${\underline{x}}_{\alpha }(t)$ and ${\overset{̅}{x}}_{\alpha }(t)$ are differentiable functions and ${\left[\dot{\tilde{x}}\left(t\right)\right]}^{\alpha }=\left[{\underline{\dot{x}}}_{\alpha }\left(t\right),{\dot{\overset{̅}{x}}}_{\alpha }\left(t\right)\right]$. If $\tilde{x}(t)$ is differentiable in the second form (ii), then ${\underline{x}}_{\alpha }(t)$ and ${\overset{̅}{x}}_{\alpha }(t)$ are differentiable functions and ${\left[\dot{\tilde{x}}\left(t\right)\right]}^{\alpha }=\left[{\dot{\overset{̅}{x}}}_{\alpha }\left(t\right),{\dot{\underline{x}}}_{\alpha }\left(t\right)\right]$.

**Proof**

Consider the following fuzzy initial value problem:

(3) $$\dot{\tilde{x}}\left(t\right)=f\left(t,\tilde{x}\left(t\right)\right),\;\;\tilde{x}\left(0\right)={\tilde{x}}_{0},$$

where *f* : *I* × *F*(*R*) → *F*(*R*) is a fuzzy function and ${\tilde{x}}_{0}\in F(R)$. Let ${\left[f(t,\tilde{x})\right]}^{\alpha }=[{\underline{f}}_{\alpha }(t,{\underline{x}}_{\alpha },{\overset{̅}{x}}_{\alpha }),\bar{f_{\alpha }}(t,{\underline{x}}_{\alpha },{\overset{̅}{x}}_{\alpha })]$. From Theorem 10, if we consider $\tilde{x}(t)$ by using the derivative in the first form (i), then the solution of problem (3) is obtained by solving the following system of ordinary differential equations:

(4) $$\begin{array}{cc} {\dot{\underline{x}}}_{\alpha }\left(t\right) & =\underline{f_{\alpha }}\left(t,{\underline{x}}_{\alpha }\left(t\right),{\overset{̅}{x}}_{\alpha }\left(t\right)\right),\;\;{\underline{x}}_{\alpha }\left(0\right)={\underline{x}}_{0\alpha },\\ {\dot{\overset{̅}{x}}}_{\alpha }\left(t\right) & =\overset{̅}{f_{\alpha }}\left(t,{\underline{x}}_{\alpha }\left(t\right),{\overset{̅}{x}}_{\alpha }\left(t\right)\right),\;\;{\overset{̅}{x}}_{\alpha }\left(0\right)={\overset{̅}{x}}_{0\alpha }, \end{array}$$

and ensuring that $\left[{\underline{x}}_{\alpha }(t),{\overset{̅}{x}}_{\alpha }(t)\right]$ and $\left[{\underline{\dot{x}}}_{\alpha }(t),{\dot{\overset{̅}{x}}}_{\alpha }\left(t\right)\right]$ are valid **α**-level sets. Moreover, if we consider $\tilde{x}(t)$ by using the derivative in the second form (ii), then the solution of problem (3) is obtained by solving the following system of ordinary differential equations,

(5) $$\begin{array}{cc} {\dot{\overset{̅}{x}}}_{\alpha }\left(t\right) & =\underline{f_{\alpha }}\left(t,{\underline{x}}_{\alpha }\left(t\right),{\overset{̅}{x}}_{\alpha }\left(t\right)\right),\;\;{\underline{x}}_{\alpha }\left(0\right)={\underline{x}}_{0\alpha },\\ {\dot{\underline{x}}}_{\alpha }\left(t\right) & =\overset{̅}{f_{\alpha }}\left(t,{\underline{x}}_{\alpha }\left(t\right),{\overset{̅}{x}}_{\alpha }\left(t\right)\right),\;\;{\overset{̅}{x}}_{\alpha }\left(0\right)={\overset{̅}{x}}_{0\alpha }, \end{array}$$

and ensuring that $\left[{\underline{x}}_{\alpha }(t),{\overset{̅}{x}}_{\alpha }(t)\right]$and $\left[{\dot{\overset{̅}{x}}}_{\alpha }\left(t\right),{\dot{\underline{x}}}_{\alpha }\left(t\right)\right]$ are valid **α**-level sets.

The integral of fuzzy function $\tilde{x}(t)$ using the Riemann integral concept can be defined as follows.

**Definition 11**

The integral of a fuzzy mapping $\tilde{x}:[a,b]\to F(R)$ is defined levelwise by

(6) $${\left[\overset{b}{\underset{a}{\int }}\tilde{x}(t)dt\right]}^{\alpha }=\left[\overset{b}{\underset{a}{\int }}{\underline{x}}_{\alpha }\left(t\right)dt,\;\overset{b}{\underset{a}{\int }}{\overset{̅}{x}}_{\alpha }\left(t\right)dt\right].$$

Note that if $\tilde{x}:[a,b]\to F(R)$ is continuous in the metric *D*, then it is integrable, that is, $\int_{\;a}^{\;b}\tilde{x}(t)dt\in F(R)$.

**Example 12**

Define the fuzzy mapping $\tilde{x}:[0,0.5]\to F(R)$ by $\tilde{x}(t)=(t,\sin\pi t,\cos\pi t)$. Then, ${\left[\int_{a}^{b}\tilde{x}(t)dt\right]}^{\alpha }=\left[\int_{0}^{0.5}\left(\;t-(1-\alpha )\sin\pi t\;\right)dt,\int_{0}^{0.5}\left(\;t+(1-\alpha )\cos\pi t\right)dt\right]=[0.125-(1-\alpha )\pi^{-1},0.125+(1-\alpha )\pi^{-1}]$; hence, $\int_{a}^{b}\tilde{x}(t)dt=(0.125,\pi^{-1})$.

## 3. Linear Fuzzy Model of HIV Infection

HIV infection can be characterized as a disease of the immune system, with progressive depletion of defensive cells, resulting in immunosuppression and susceptibility to opportunistic infections. CD4+ T-cells, CTLs, and the virus particles play important roles in HIV infection. CD4+ T-cells are a fundamental component of the human immune response system. These cells can be considered “messengers” or the command centers of the immune system, and they signal other immune cells that an invader is to be fought. The immune response cells, or cytotoxic lymphocytes (CTLs), are the cells that respond to this message and set out to eliminate infection by killing infected cells. HIV can infect a number of cells in the body however, its main target is the CD4+ T-cells. HIV enters these cells by a complex process and begins to replicate, then the new virus particles are released by bursting the infected cells. CD4+ T-cells are generated from sources within the body and are lost either by having finite life span or by bursting during the proliferation of HIV, which leads to a drop in the number of these cells, after infection and an accelerated decrease during the later stages of the disease that signals the onset AIDS. In accordance with experimental findings, too high a level of HIV impairs establishment of a lasting CTL response. This is a delicate task, since CD4+ T-cell population, which plays an essential role in stimulation of immune response, depletes dramatically with raising the HIV load. The rate of CD4+ T-cells depletion varies greatly from patient to patient, depending on the strength or weakness of the immune system. More precisely, a stronger immune system leads to a lower rate of CD4+ T-cells depletion and *vice versa*. We have a similar argument about the proliferation rate of HIV particles. Therefore, the levels of the immune cells as well as the HIV viral load during the different stages of the disease can be considered as fuzzy quantities. According to these descriptions, the interaction of HIV with the immune system can be modeled by a system of linear differential equations with fuzzy parameters as follows:

(7) $$\begin{array}{c} \dot{\tilde{x}}=\tilde{\lambda }\Theta \tilde{\sigma }⊗\tilde{x}\Theta \tilde{c}⊗\tilde{v},\\ \dot{\tilde{v}}=\tilde{k}⊗\tilde{v}\Theta \tilde{a}⊗\tilde{z},\\ \dot{\tilde{z}}=\tilde{h}⊗\tilde{x}\Theta \tilde{\tau }⊗\tilde{v}, \end{array}$$

where the fuzzy functions $\tilde{x}(t)$, $\tilde{z}(t)$, and $\tilde{v}(t)$ indicate the level of CD4+ T-cells, CTLs, and the HIV viral load at time* t*, respectively. Most of the terms in the model have straightforward interpretations as follows.

 The first equation in (7) represents the dynamics of the concentration of CD4+ T-cells. The CD4+ T-cells are produced from a source, such as the thymus, at a constant rate $\tilde{\lambda }$. Here, we have assumed that CD4+ T-cells have a finite life-span and die at a rate $\tilde{\sigma }$ per cell. Therefore, the number of these cells, which are lost due to natural death, is represented through the loss term $\tilde{\sigma }⊗\tilde{x}$ in the first equation. Moreover, the CD4+ T-cell population is lost through infection by a virus particle at a rate of $\tilde{c}$, and so the term $\tilde{c}⊗\tilde{v}$ models the rate that free viruses destroy CD4+ T-cells. The second equation in (7) depicts the rate of change in the virus population. An HIV particle uses a host cell to replicate itself and thus proliferates with a growth rate $\tilde{k}$. Thus, the total amount of produced viruses is given by the term $\tilde{k}⊗\tilde{v}$. Infected cells are killed by CTLs, and hence viruses are lost through an immune response. Assuming that a CTL eliminates the virus particles at a rate $\tilde{a}$, the number of virus particles eliminated by the immune response is given by the term $\tilde{a}⊗\tilde{z}$. The third equation in (7) describes the dynamics of CTLs during HIV infection. A CD4+ T-cell stimulates CTLs to proliferate at a rate $\tilde{h}$. Therefore, CD4+ T-cells effect on proliferation of CTLs is expressed by the term $\tilde{h}⊗\tilde{x}$. The term $\tilde{\tau }⊗\tilde{v}$ takes into account loss of CTLs due to increasing the HIV viral load where $\tilde{\tau }$ is the rate at which the virus-induced impairment of CD4+ T-cell function occurs.

**Figure 4:**
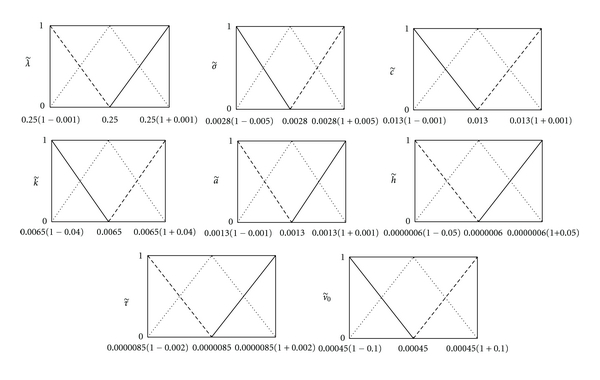
The values of model parameters corresponding to patients **W** (- -), **M** (*⋯*), and **S** (—).

 In this paper, a patient with respect to the strength or weakness of its immune system is considered as a patient with the weak, moderate, or strong immune system and is indicated by *W*, *M*, or *S*, respectively. The initial condition of $\tilde{v}(0)={\tilde{v}}_{0}$ varies in different patients. For that reason, a primary response is provoked when the immune system encounters HIV for the first time and, in this stage, a number of viruses depending on the strength or weakness of the immune system are eliminated, and the proposed model describes the changes in the immune cells level and the viral load after this stage which is called the secondary immune response. Therefore, a stronger immune response implies a lower ${\tilde{v}}_{0}$ and *vice versa*. The values of the model parameters and ${\tilde{v}}_{0}$ corresponding to patients *W*, *M*, and *S* are shown in Figure 4 as triangular fuzzy numbers. These parameters were chosen to be consistent with biological plausibility. Moreover, we assume that, at time *t* = 0, the level of CD4+ T-cells is normal and there is no CTL-mediated immune response in all patients, that is, $\tilde{x}(0)={\tilde{x}}_{0}=(100,0)$ and $\tilde{z}(0)={\tilde{z}}_{0}=(0,0)$. We must note that $\tilde{x}(t)$ denotes CD4+ T-cells level in percentage at time *t*. The derivative in the second form (ii) leads to solutions with decreasing length of their support which leads us to the conclusion that the uncertainty decreases with the time lapse which is not consistent with real situation. Moreover, the existence of these solutions implies that the initial conditions should be fuzzy. Therefore, we consider only the solutions with the derivative in the first forms (i) which are more consistent with real situation. Consequently, as mentioned in Section 2, the fuzzy model (7) is transformed to the following system of ordinary differential equations (ODEs):

(8) $$\;\begin{array}{c} {\dot{\overset{̅}{x}}}_{\alpha }\left(t\right)={\overset{̅}{\lambda }}_{\alpha }-{\underline{\sigma }}_{\alpha }{\underline{x}}_{\alpha }\left(t\right)-{\underline{c}}_{\alpha }{\underline{v}}_{\alpha }\left(t\right),\\ {\dot{\underline{x}}}_{\alpha }\left(t\right)={\underline{\lambda }}_{\alpha }-{\overset{̅}{\sigma }}_{\alpha }{\overset{̅}{x}}_{\alpha }\left(t\right)-{\overset{̅}{c}}_{\alpha }{\overset{̅}{v}}_{\alpha }\left(t\right),\\ {\dot{\overset{̅}{v}}}_{\alpha }\left(t\right)={\overset{̅}{k}}_{\alpha }{\overset{̅}{v}}_{\alpha }\left(t\right)-{\underline{a}}_{\alpha }{\underline{z}}_{\alpha }\left(t\right),\\ {\dot{\underline{v}}}_{\alpha }\left(t\right)={\underline{k}}_{\alpha }{\underline{v}}_{\alpha }\left(t\right)-{\overset{̅}{a}}_{\alpha }{\overset{̅}{z}}_{\alpha }\left(t\right),\\ {\dot{\overset{̅}{z}}}_{\alpha }\left(t\right)={\overset{̅}{h}}_{\alpha }{\overset{̅}{x}}_{\alpha }\left(t\right)-{\underline{\tau }}_{\alpha }{\underline{v}}_{\alpha }\left(t\right),\\ {\dot{\underline{z}}}_{\alpha }\left(t\right)={\underline{h}}_{\alpha }{\underline{x}}_{\alpha }\left(t\right)-{\overset{̅}{\tau }}_{\alpha }{\overset{̅}{v}}_{\alpha }\left(t\right),\\ {\overset{̅}{x}}_{\alpha }\left(0\right)={\overset{̅}{x}}_{0\alpha },\;\;{\underline{x}}_{\alpha }\left(0\right)={\underline{x}}_{0\alpha },\\ {\overset{̅}{v}}_{\alpha }\left(0\right)={\overset{̅}{v}}_{0\alpha },\;\;{\underline{v}}_{\alpha }\left(0\right)={\underline{v}}_{0\alpha },\\ {\overset{̅}{z}}_{\alpha }\left(0\right)={\overset{̅}{z}}_{0\alpha },\;\;{\underline{z}}_{\alpha }\left(0\right)={\underline{z}}_{0\alpha }. \end{array}$$

For each *α* ∈ [0,1], the ODEs (8) are linear; hence, the exact solutions in discrete times are obtained using the *ode45* code in MATLAB. However, it would be appropriate to propose explicit solutions as a function of *α* and *t*. The next section is devoted to this topic.

### 3.1. The Approximate Explicit Solutions Based on a Fitting Method

The proposed method is based on the fact that a linear combination of suitable functions of **α** and *t* can generate the best fit to the exact values obtained by the *ode45* in the least squares sense. The following discussion shows that these functions can be exponential. The ODEs (8) can be written in a matrix form as:

(9) $$\begin{array}{c} {\dot{X}}_{\alpha }\left(t\right)=A_{\alpha }X_{\alpha }\left(t\right)+B_{\alpha },\\ X_{\alpha }\left(0\right)=X_{0\alpha }. \end{array}$$

By the variation of constants formula for ordinary differential equations, the solution of the initial value problem (9) is *X*_*α*_(*t*) = *e* ^*A*_*α*_*t*^ *X*_0*α*_ + ∫_0_^*t*^ *e* ^*A*_*α*_  (*t*−*τ*)^ *B*_*α*_ *dτ*. Since the six-dimensional matrix *A*_*α*_ depends on **α**, the calculation of *e* ^*A*_*α*_*t*^ becomes difficult. But this matrix can be written as *A*_*α*_ = *A*_1_ + (1 − *α*)*A*_2_, where *A*_1_ and *A*_2_ are **α*-*independent matrices. We have *e* ^*A*_*α*_*t*^ = *e* ^*A*_1_*t*^ *e* ^(1−*α*)*A*_2_*t*^ + *O*(*t*), where *O*(*t*) is a function that lim⁡_*t*→0_ *O*(*t*)/*t* = 0. Assuming that *ℓ*_*j*_s and *ς*_*j*_ *s*, *j* = 1,…, 6, are eigenvalues of *A*_1_ and *A*_2_, respectively, there are invertible matrices *P* and *Q* such that *A*_1_ = *PD*_1_ *P* ^−1^ and *A*_2_ = *QD*_2_ *Q* ^−1^, where *D*_1_ = diag⁡(*ℓ*_1_,…, *ℓ*_6_) and *D*_2_ = diag⁡(*ς*_1_,…, *ς*_6_). Therefore, for small *t*,

(10) $$\begin{array}{cc} e^{A_{\alpha }t} & \approx P\left(\begin{array}{cccc} e^{\ell_{1}t} & 0 & \cdots & 0\\ 0 & e^{\ell_{2}t} & \cdots & \vdots \\ \vdots & \vdots & \ddots & 0\\ 0 & \cdots & 0 & e^{\ell_{6}t} \end{array}\right)P^{\;-1}\\ & \;\times Q\left(\begin{array}{cccc} e^{(1-\alpha )\zeta_{1}t} & 0 & \cdots & 0\\ 0 & e^{(1-\alpha )\zeta_{2}t} & \cdots & \vdots \\ \vdots & \vdots & \ddots & 0\\ 0 & \cdots & 0 & e^{(1-\alpha )\zeta_{6}t} \end{array}\right)Q^{\;-1}. \end{array}$$

As a result, the fitting functions are chosen as *e* ^(*ℓ*_*i*_+(1−*α*)*ς*_*j*_)*t*^, *i*, *j* = 1,…, 6. Therefore, by choosing the numbers *ℓ*_*j*_, *j* = 1,…, *n*, and *ς*_*i*_, *i* = 1,…, *m*, where *n*, *m* ∈ {1,2,…, 6}, an approximate solution can be found in the following form:

(11) $$S_{\alpha }\left(t\right)\approx KE_{\alpha }\left(t\right),$$

where $S_{\alpha }(t)={\left[{\overset{̅}{x}}_{\alpha }(t),{\underline{x}}_{\alpha }(t),{\overset{̅}{v}}_{\alpha }(t),{\underline{v}}_{\alpha }(t)\right]}^{T}$, *E*_*α*_(*t*) = [*e* ^(*ℓ*_1_+(1−*α*)*ς*_1_)*t*^,…, *e* ^(*ℓ*_1_+(1−*α*)*ς*_*m*_)*t*^,…, *e* ^(*ℓ*_*n*_+(1−*α*)*ς*_1_)*t*^,…, *e* ^(*ℓ*_*n*_+(1−*α*)*ς*_*m*_)*t*^]^*T*^ and *K* = [*k*_*ij*_]_4×*nm*_ denotes the coefficients matrix that can be found using the *lsqnonlin* code of the optimization toolbox in MATLAB. Obviously, ${\overset{̅}{z}}_{\alpha }(t)$ and ${\underline{z}}_{\alpha }(t)$ are obtained from the 5th and the 6th equations in (8) using the approximate values ${\overset{̅}{x}}_{\alpha }(t)$, ${\underline{x}}_{\alpha }(t)$, ${\overset{̅}{v}}_{\alpha }(t)$, and ${\underline{v}}_{\alpha }(t)$.

### 3.2. Dynamic Behavior of the Immune Cells Level and the Viral Load in Patient *W*

**Figure 5:**
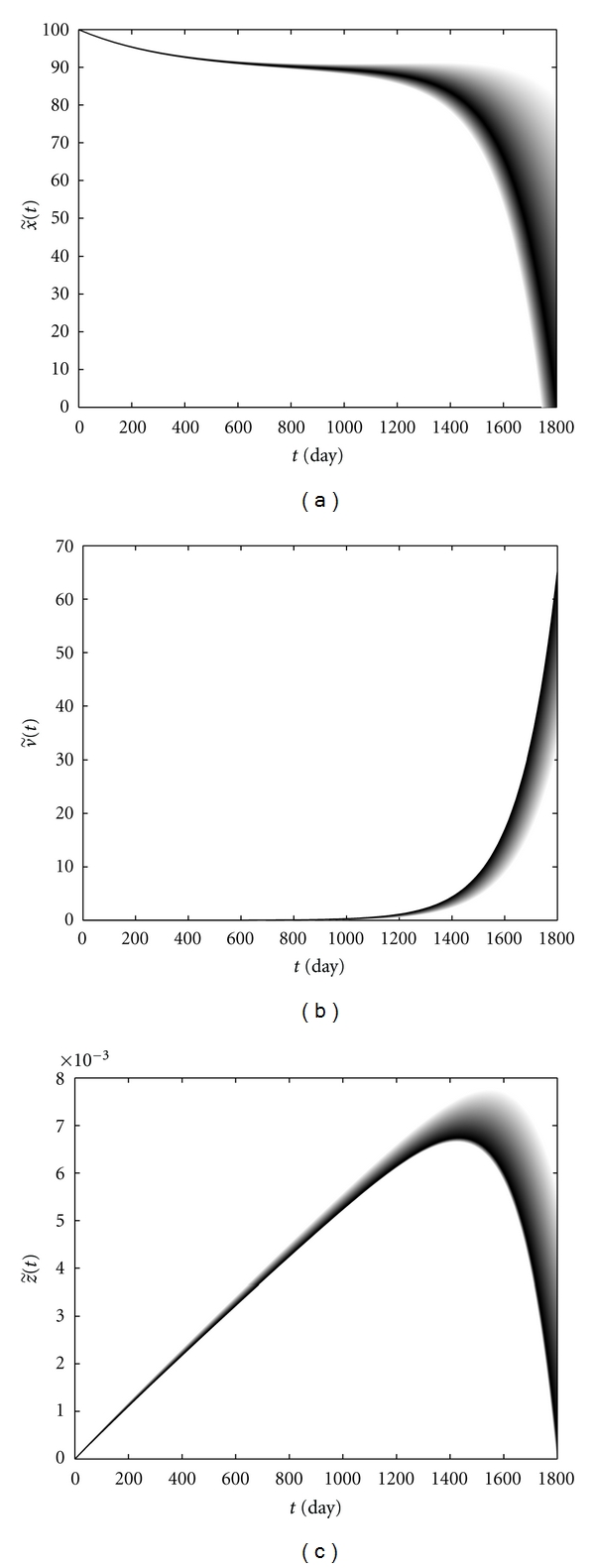
CD4+ T-cells level (a), the viral load (b), and CTLs level (c) versus time in patient *W. *

Figure 5 shows the level of immune cells and the HIV viral load of patient *W* during the time interval [0, 1800]. The darker color shows the curve with the higher possibility.  Figure 5(a) shows that the gradual declines in CD4+ T-cells level correspond to the low possibilities, while the rapid declines and the progression to full blown AIDS after a gradual decay, have the high possibilities of occurrence.  Figure 5(b) shows a rapid increase in the viral load during the later stages of the disease. Moreover, we observe that, at each time, a higher viral load corresponds to a higher possibility.  Figure 5(c) shows a clear correlation between CTLs level in the blood and HIV progression. As the viral load increases upon initial infection, CTLs increase in order to decrease the virus. But ultimately the level of these cells begins to decrease, which is due to virus-induced impairment of CD4+ T-cell function, with the high possibilities after about the 1450th day. Besides, a lower CTLs level has a higher possibility of occurrence and *vice versa*. From Figures 5(a) and 5(b), there is an inverse correlation between the HIV viral load and the level of CD4+ T-cells. Following the proposed method in Section 3.1, choose the eigenvalues of the corresponding matrices *A*_1_ and *A*_2_ as

(12) $$\begin{array}{cc} \left(\varsigma_{1},\varsigma_{2}\right) & =\left(-0.2600\;\times \;10^{-3},\;0.6537\;\times \;10^{-11}\right),\\ \left(\ell_{1},\ell_{2},\ell_{3},\ell_{4},\ell_{5}\right) & =(-\;0.2801\;\times \;10^{-2},\;0.2801\;\times \;10^{-2},\;\\ & \;\;-0.2145\;\times \;10^{-6},\\ & \;\;-\;0.1128\;\times \;10^{-6},0.6760\;\times \;10^{-2}). \end{array}$$

Then, we have an approximate explicit solution in the form of (11) where the corresponding coefficients matrix *K* is

(13) $$K=\left[\begin{array}{cccccccccc} 0.6684 & 10.1635 & -0.2245 & 0.2229 & -0.7100 & 45.2900 & -0.7028 & 45.2924 & -0.0009 & 0.0005\\ -3.1406 & 13.9779 & 0.1989 & -0.1974 & 0.6763 & 43.8973 & 0.6832 & 43.8994 & 0.0004 & -0.0008\\ -0.0123 & 0.0109 & -0.0002 & 0.0002 & 0.0044 & -0.0038 & 0.0044 & -0.0038 & -0.0000 & 0.0003\\ 0.8273 & -0.8222 & 0.0219 & -0.0217 & -0.1624 & 0.1608 & -0.1623 & 0.1609 & 0.0004 & -0.0001 \end{array}\right].$$

**Figure 6:**
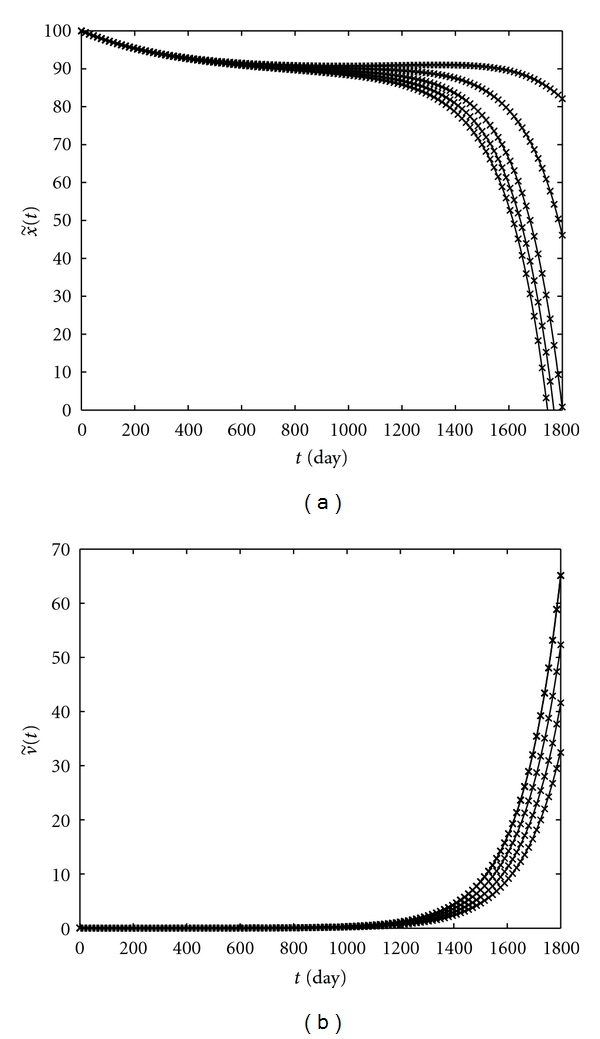
Exact (—) and approximate (×) CD4+ T-cells level (a) and the viral load (b) in patient *W*.

A comparison between the exact and approximate solutions which is shown in Figure 6 confirmed the effectiveness of this approach.

### 3.3. Dynamic Behavior of the Immune Cells Level and the Viral Load in Patient *S*

**Figure 7:**
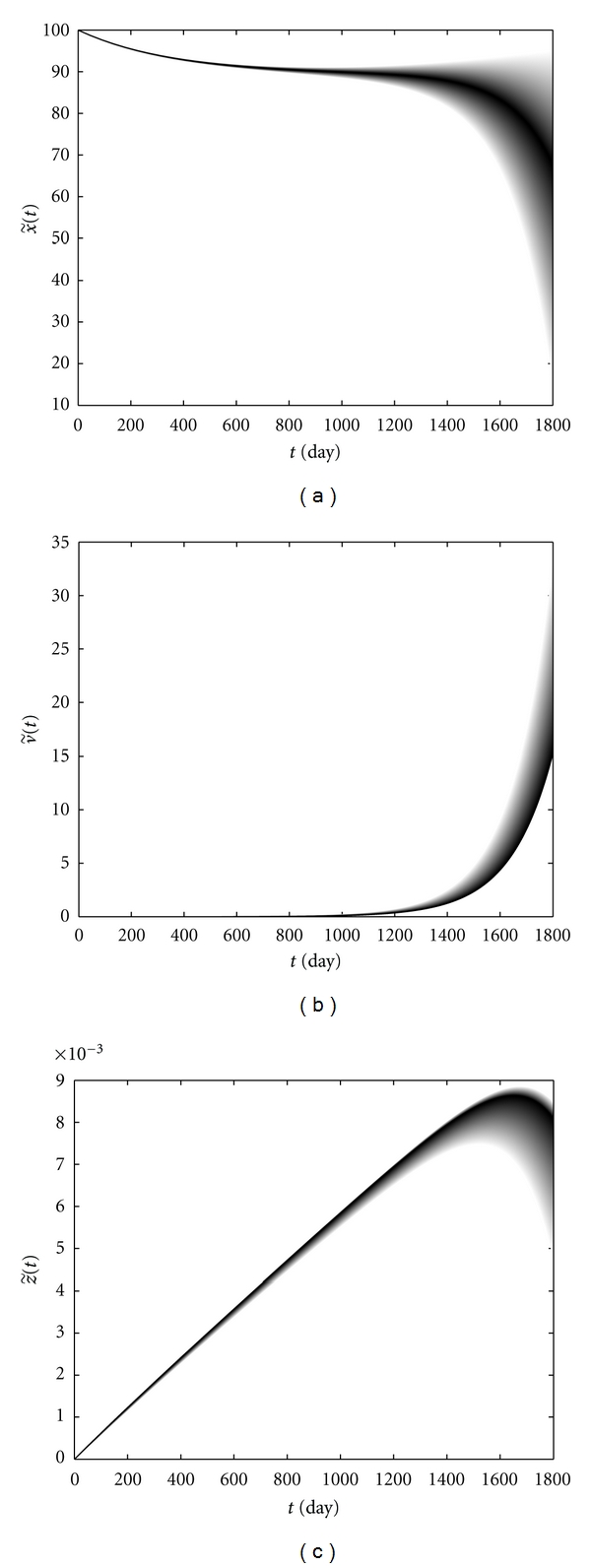
CD4+ T-cells level (a), the viral load (b), and CTLs level (c) versus time in patient *S*.

Figure 7 shows the changes in the immune cells level and the viral load in patient *S*. With respect to Figure 7(a), CD4+ T-cells level decreases gradually during the 1800 days from infection with the high possibilities. Moreover, an increase in CD4+ T-cells as well as the progression to AIDS arises with a low possibility. The HIV viral load is low and a lower viral load has a higher possibility of occurring, as shown in Figure 7(b). A high CD4+ T-cell count and a low HIV viral load lead to establishment of a lasting CTL response which is shown in Figure 7(c). A high HIV viral load and a low CD4+ T-cells level impair the immune response where this arises with the low possibilities as shown in Figure 7 as the light curves. The selected numbers *ℓ*_*i*_ *s* and *ς*_*j*_ *s* and the corresponding coefficients matrix *K* representing the approximate explicit solutions in the form of (11) are as

(14) $$\begin{array}{c} \left(\varsigma_{1},\varsigma_{2}\right)=\left(0.2600\times 10^{-3},0.6537\times 10^{-11}\right),\\ \left(\ell_{1},\ell_{2},\ell_{3},\ell_{4},\ell_{5}\right)=\left(-\;0.2800\times 10^{-2},\;0.2800\times 10^{-2},\;-0.1159\times 10^{-6},\;0.6240\times 10^{-2},-0.2378\times 10^{-6}\right),\\ K=\left[\begin{array}{cccccccccc} -3.3203 & 13.9175 & 0.1287 & -0.1265 & 0.7662 & 43.9293 & 0.0003 & -0.0006 & 0.7723 & 43.9412\\ 0.8303 & 9.7266 & -0.1431 & 0.1412 & -0.8060 & 45.5176 & -0.0008 & 0.0005 & -0.7996 & 45.5300\\ 0.3895 & -0.3902 & 0.0110 & -0.0109 & -0.0724 & 0.0725 & 0.0004 & -0.0002 & -0.0724 & 0.0726\\ 0.0076 & -0.0099 & 0.0002 & -0.0002 & -0.0051 & 0.0063 & -0.0000 & 0.0002 & -0.0051 & 0.0063 \end{array}\right]. \end{array}$$

### 3.4. Dynamic Behavior of the Immune Cells and the Viral Load in Patient *M*

**Figure 8:**
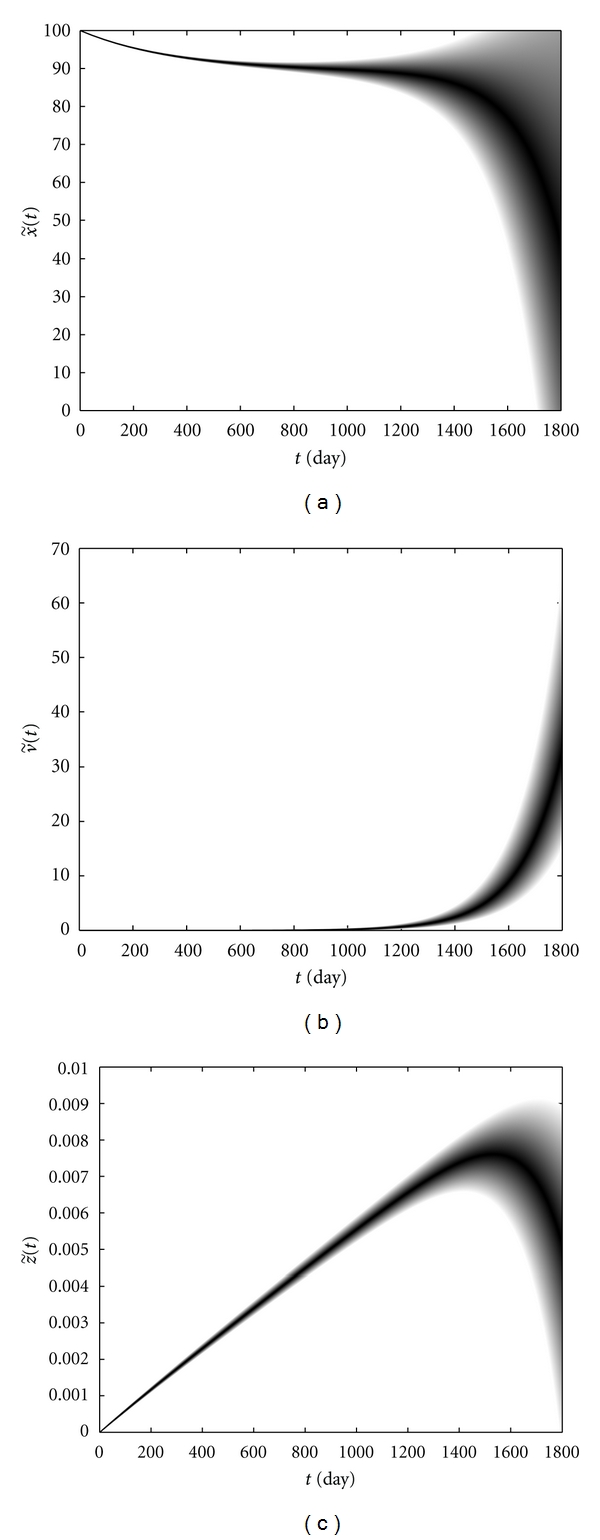
CD4+ T-cells level (a), the viral load (b), and CTLs level (c) versus time in patient *M*.

Figure 8 shows the changes in the immune cells level and the viruses in patient *M*. From Figure 8(a), the uncertainty of CD4+ T-cells level increases and new possibilities, varying fromm an increment to normal level to rapid progression to full blown AIDS, arise after a gradual decay. But the most possible scenario is between where the level of these cells decreases at a moderate rate. A moderate viral load occurs with a high possibility, as shown in Figure 8(b). A moderate CD4+ T-cells level as well as a moderate viral load implies a moderate CTLs level, as shown in Figure 8(c). Besides, from this figure, a high (low) viral load and a low (high) CD4+ T-cells number decrease (increase) CTLs level, and this happens with a low possibility.

 Here, a representation for solutions is given in the form of (11) where

(15) $$\begin{array}{c} \left(\ell_{1},\ell_{2},\ell_{3},\ell_{4},\ell_{5}\right)=\left(-0.2800\times 10^{-2},\;0.2800\times 10^{-2},\;0.2257\times 10^{-6},-0.1143\times 10^{-6},\;0.6500\times 10^{-2}\right),\\ \left(\varsigma_{1},\varsigma_{2},\varsigma_{3}\right)=\left(0,\;-0.2600\times 10^{-3},\;0.2600\times 10^{-3}\right),\\ K=\left[\begin{array}{ccccccccccccccc} 45.7977 & -20.3001 & -14.9203 & 0.8002 & -0.6250 & -0.1772 & 26.5114 & 9.6039 & 8.5734 & 26.5188 & 9.6169 & 8.5785 & -0.0004 & -0.0006 & 0.0006\;\\ 25.4227 & -12.6502 & -2.1878 & 0.4622 & -0.0933 & -0.3705 & 31.2491 & 8.3945 & 5.0396 & 31.2549 & 8.4097 & 5.0415 & -0.0000 & 0.0005 & -0.0008\;\\ -0.2693 & -0.3082 & 0.5693 & -0.0159 & -0.0016 & 0.0174 & -0.0068 & 0.0869 & -0.0772 & -0.0069 & 0.0869 & -0.0773 & -0.0003 & 0.0001 & 0.0005\;\\ -0.0517 & 0.2515 & -0.2007 & -0.0035 & 0.0074 & -0.0040 & -0.0033 & -0.0363 & 0.0406 & -0.0033 & -0.0363 & 0.0406 & -0.0002 & 0.0004 & 0.0000\; \end{array}\right]. \end{array}$$

### 3.5. A Comparison between the Immune Cells Level and the Viral Load in Patients *W*, *M*, and *S*

**Figure 9:**
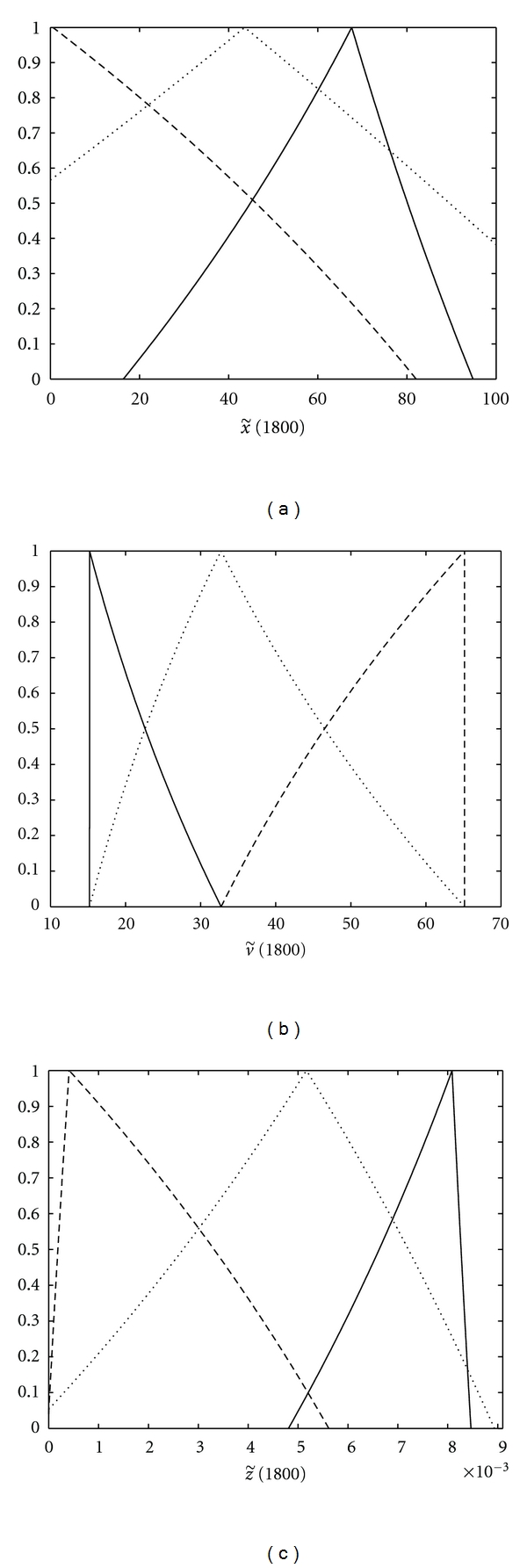
The fuzzy numbers indicating CD4+ T-cells level (a), the viral load (b), and CTLs level (c) on the 1800th day in patients *W* (- -), *M* (*⋯*) and, *S* (—).

From Figures 5(b), 7(b), and 8(b), there is an inverse correlation between the viral load and the immune system strength. CD4+ T-cell and CTL levels in patient *S* are more than the level of these cells in patient *W*, as shown in Figures 5 and 7. A high viral load in patient *W* leads to a virus-induced impairment of CD4+ T-cell function. Therefore, CTLs level in this patient is less than the level of these cells in patient *S*, as shown in Figures 5(c) and 7(c). A comparison between CTLs level in patients *M* and *S* shows that it is possible that CTLs level in patient *M* be slightly more than the level of these cells in patient *S*, which can be due to this fact that patient *M* has more antigens that are required to stimulate CTLs (see Figures 7(b) and 8(b)), and, thus, a higher level of CD4+ T-cells in patient *M* is possible as shown in Figures 7(a) and 8(a).  Figure 9 shows CD4+ T-cell and CTL levels and the viral load of patients *W*, *M*, and *S* on the 1800th day. Here, we perform a comparison between the immune cells level and the HIV viral load of patients based on their weighted center of gravity (WCOG).

 For this end, the WCOG of $\tilde{x}(t)$, $\tilde{v}(t)$, and $\tilde{z}(t)$ is denoted by *x*(*t*), *v*(*t*), and *z*(*t*), respectively; hence,

(16) $$\begin{array}{c} x\left(t\right)=\overset{1}{\underset{0}{\int }}\alpha \left({\overset{̅}{x}}_{\alpha }\left(t\right)+{\underline{x}}_{\alpha }\left(t\right)\right)d\alpha ,\\ v\left(t\right)=\overset{1}{\underset{0}{\int }}\alpha \left({\overset{̅}{v}}_{\alpha }\left(t\right)+{\underline{v}}_{\alpha }\left(t\right)\right)d\alpha ,\\ z\left(t\right)=\overset{1}{\underset{0}{\int }}\alpha \left({\overset{̅}{z}}_{\alpha }\left(t\right)+{\underline{z}}_{\alpha }\left(t\right)\right)d\alpha . \end{array}$$

With respect to (11), an approximate explicit formula for the WCOG of $\tilde{x}(t)$ and $\tilde{v}(t)$ is given as *S*(*t*) ≈ *NF*(*t*), where *S*(*t*) = [*x*(*t*), *v*(*t*)]^*T*^and *N* is a matrix with two rows that its first and second rows are obtained by summing the first two rows and the last two rows of the corresponding coefficients matrix *K*, respectively. Moreover,

(17) $$\begin{array}{cc} F\left(t\right) & =\overset{1}{\underset{0}{\int }}\alpha \;E_{\alpha }\left(t\right)d\alpha \\ & =[\frac{e^{\ell_{1}t}}{\varsigma_{1}t}\left(\frac{e^{\varsigma_{1}t}}{\varsigma_{1}t}-\frac{1}{\varsigma_{1}t}-1\right),\ldots ,\;\\ & \;\;\frac{e^{\ell_{1}t}}{\varsigma_{m}t}\left(\frac{e^{\varsigma_{m}t}}{\varsigma_{m}t}-\frac{1}{\varsigma_{m}t}-1\right),\ldots ,\\ & \;\;\frac{e^{\ell_{n}t}}{\varsigma_{1}t}\left(\frac{e^{\varsigma {\;}_{1}t}}{\varsigma_{1}t}-\frac{1}{\varsigma_{1}t}-1\right),\ldots ,\\ & \;\;{\frac{e^{\ell_{n}t}}{\varsigma_{m}t}\left(\frac{e^{\varsigma_{m}t}}{\varsigma_{m}t}-\frac{1}{\varsigma_{m}t}-1\right)]}^{T}. \end{array}$$

**Figure 10:**
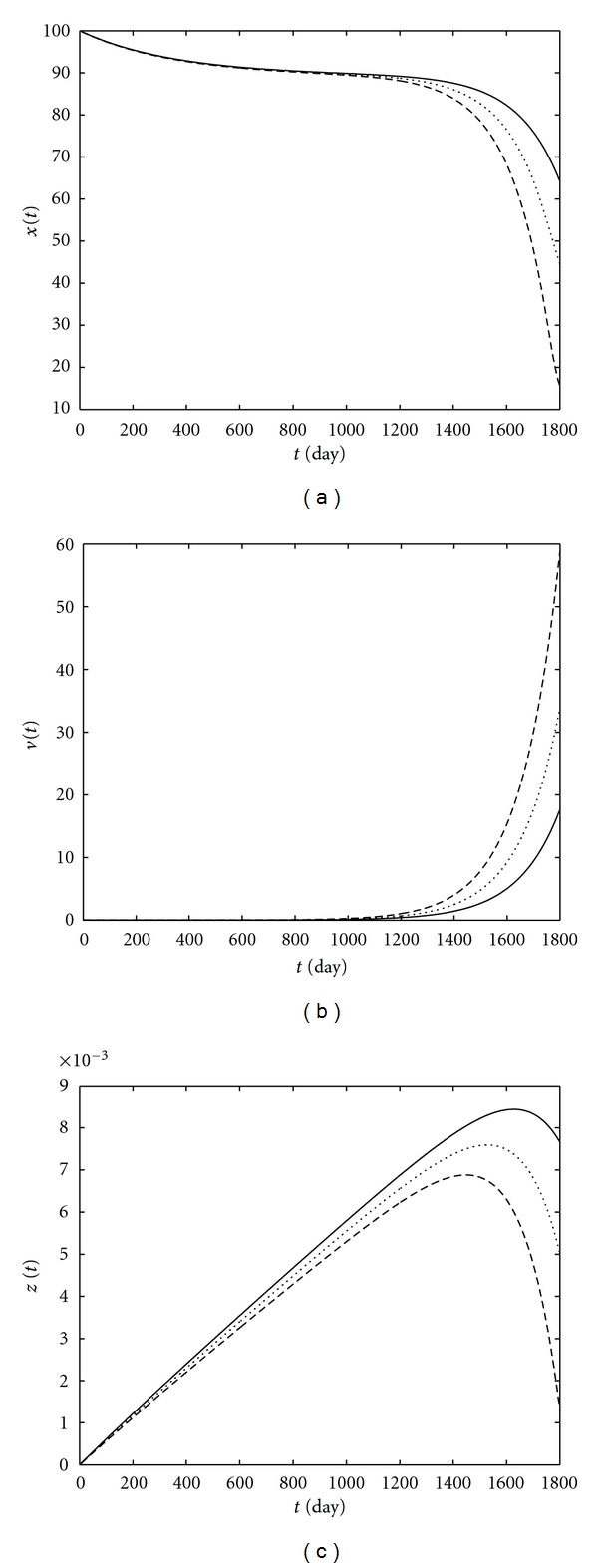
The WCOG of CD4+ T-cells level (a), the viral load (b) and CTLs level (c) versus time in patients *W* (- -), *M* (*⋯*), and *S* (—).

The WCOG of $\tilde{x}(t)$, $\tilde{v}(t)$, and $\tilde{z}(t)$ corresponding to patients *W*, *M*, and *S* is shown in Figure 10. From Figures 10(a) and 10(c), CD4+ T-cells and CTLs level are proportional to the strength of patient's immune system such that a stronger immune system leads to a higher level of these cells. Moreover, there is an inverse correlation between the viral load and the strength of the immune system as shown in Figure 10(b).

## 4. Fuzzy Optimal Control Problem

In this section, we formulate a fuzzy optimal control problem that identifies the parameter $\tilde{k}$ in (8), with a function of the fuzzy control variable $\tilde{u}$. In particular, we will replace the parameter $\tilde{k}$ with the function $\tilde{k}\;\Theta_{H}\;\tilde{u}$. This choice then identifies the control variable $\tilde{u}(t)$ with the rate of inhibition of virus reproduction, which is modeled as a simple function of drug dosage. Therefore, we have the fuzzy set control differential equations (FSCDEs) as

(18) $$\begin{array}{c} \dot{\tilde{x}}=\tilde{\lambda }\;\Theta \;\tilde{\sigma }⊗\tilde{x}\;\Theta \;\tilde{c}⊗\tilde{v},\\ \dot{\tilde{v}}=\left(\tilde{k}{\;\Theta }_{H}\;\tilde{u}\right)⊗\tilde{v}\;\Theta \;\tilde{a}⊗\tilde{z},\\ \dot{\tilde{z}}=\tilde{h}⊗\tilde{x\;}\Theta \;\tilde{\tau }⊗\tilde{v}. \end{array}$$

This paper aims to propose a drug regimen that minimizes both the viral load and the drug costs. Here, we assume that the cost of the treatment is proportional to ${\tilde{u}}^{2}(t)$ at time *t*. Therefore, the fuzzy functional $\tilde{J}(\tilde{v},\tilde{u})=\int_{t_{0}}^{t_{f}}(\tilde{w}⊗\tilde{v}(t)⊕{\tilde{u}}^{2}(t))dt$ should be minimized, where the positive fuzzy number $\tilde{w}$ is used to set the relative importance between minimizing the viral load and the systemic cost to the body. Let *T* = [*t*_0_,  *t*_*f*_], and assume *C*(*T*) be the set of all continuous fuzzy number valued functions on *T*. Assuming that the minimum and the maximum of allowable drug dosage are denoted by fuzzy numbers ${\tilde{u}}^{\min}$ and ${\tilde{u}}^{\max}$, respectively, then we are seeking a ${\tilde{u}}^{*}\in \tilde{U}$ such that $\tilde{J}\left({\tilde{v}}^{*},{\tilde{u}}^{*}\right)\tilde{\leq }\;\tilde{J}\left(\tilde{v},\tilde{u}\right)$, for all $\tilde{u}\in \tilde{U}$, where $\tilde{U}=\{\;\tilde{u}\in C\left(T\right):{\tilde{u}}^{\min}\tilde{\leq }\;\;\tilde{u}\left(t\right)\;\tilde{\leq }\;{\tilde{u}}^{\max}$, for all *t* ∈ *T*}. By using the derivative in the first form (i), the FSCDEs (18) is converted to the following control system:

(19) $$\begin{array}{c} {\dot{\overset{̅}{x}}}_{\alpha }\left(t\right)={\overset{̅}{\lambda }}_{\alpha }-{\underline{\sigma }}_{\alpha }{\underline{x}}_{\alpha }\left(t\right)-{\underline{c}}_{\alpha }{\underline{v}}_{\alpha }\left(t\right),\\ {\dot{\underline{x}}}_{\alpha }\left(t\right)={\underline{\lambda }}_{\alpha }-{\overset{̅}{\sigma }}_{\alpha }{\overset{̅}{x}}_{\alpha }\left(t\right)-{\overset{̅}{c}}_{\alpha }{\overset{̅}{v}}_{\alpha }\left(t\right),\\ {\dot{\overset{̅}{v}}}_{\alpha }\left(t\right)=\left({\overset{̅}{k}}_{\alpha }-{\overset{̅}{u}}_{\alpha }\left(t\right)\right){\overset{̅}{v}}_{\alpha }\left(t\right)-{\underline{a}}_{\alpha }{\underline{z}}_{\alpha }\left(t\right),\\ {\dot{\underline{v}}}_{\alpha }\left(t\right)=\left({\underline{k}}_{\alpha }-{\underline{u}}_{\alpha }\left(t\right)\right){\underline{v}}_{\alpha }\left(t\right)-{\overset{̅}{a}}_{\alpha }{\overset{̅}{z}}_{\alpha }\left(t\right),\\ {\dot{\overset{̅}{z}}}_{\alpha }\left(t\right)={\overset{̅}{h}}_{\alpha }{\overset{̅}{x}}_{\alpha }\left(t\right)-{\underline{\tau }}_{\alpha }{\underline{v}}_{\alpha }\left(t\right),\\ {\dot{\underline{z}}}_{\alpha }\left(t\right)={\underline{h}}_{\alpha }{\underline{x}}_{\alpha }\left(t\right)-{\overset{̅}{\tau }}_{\alpha }{\overset{̅}{v}}_{\alpha }\left(t\right),\\ {\overset{̅}{x}}_{\alpha }\left(0\right)={\overset{̅}{x}}_{0\alpha },\;\;{\underline{x}}_{\alpha }\left(0\right)={\underline{x}}_{0\alpha },\\ {\overset{̅}{v}}_{\alpha }\left(0\right)={\overset{̅}{v}}_{0\alpha },\;\;{\underline{v}}_{\alpha }\left(0\right)={\underline{v}}_{0\alpha },\\ {\overset{̅}{z}}_{\alpha }\left(0\right)={\overset{̅}{z}}_{0\alpha },\;\;{\underline{z}}_{\alpha }\left(0\right)={\underline{z}}_{0\alpha }. \end{array}$$

With respect to Definition 5, a fuzzy function ${\tilde{u}}^{*}\in \tilde{U}$ is viewed as an optimal solution, if, for each *α* ∈ [0,1], the pair $({\underline{u}}_{\alpha }^{*},{\overset{̅}{u}}_{\alpha }^{*})\in U$ minimizes the functionals ${\bar{\left[\int_{t_{0}}^{t_{f}}(\tilde{w}⊗\tilde{v}(t)⊕{\tilde{u}}^{2}(t))dt\right]}}_{\alpha }=\int_{t_{0}}^{t_{f}}\left({\overset{̅}{w}}_{\alpha }{\overset{̅}{v}}_{\alpha }(t)+{\overset{̅}{u}}_{\alpha }^{2}(t\right))dt$ and ${\underline{\left[\int_{t_{0}}^{t_{f}}(\tilde{w}⊗\tilde{v}(t)⊕{\tilde{u}}^{2}(t))dt\right]}}_{\alpha }=\int_{t_{0}}^{t_{f}}\left({\underline{w}}_{\alpha }{\underline{v}}_{\alpha }(t)+{\underline{u}}_{\alpha }^{2}(t\right))dt$ simultaneously, where ${\overset{̅}{v}}_{\alpha }(t)$ and ${\underline{v}}_{\alpha }(t)$ are the solution of ODEs (19) corresponding to control pair $\left({\underline{u}}_{\alpha },{\overset{̅}{u}}_{\alpha }\right)$, and *U* is the set of all measurable control pairs $\left({\underline{u}}_{\alpha },{\overset{̅}{u}}_{\alpha }\right)$ that ${\underline{u}}_{\alpha }^{\min}\leq {\underline{u}}_{\alpha }\left(t\right)\leq {\underline{u}}_{\alpha }^{\max},\;{\overset{̅}{u}}_{\alpha }^{\min}\leq {\overset{̅}{u}}_{\alpha }\left(t\right)\leq {\overset{̅}{u}}_{\alpha }^{\max}$, for all *t* ∈ *T*. Therefore, we restrict our attention to optimizing the functional $J({\overset{̅}{v}}_{\alpha },{\underline{v}}_{\alpha },{\overset{̅}{u}}_{\alpha },{\underline{u}}_{\alpha })=$ $\int_{t_{0}}^{t_{f}}\left({\underline{w}}_{\alpha }{\underline{v}}_{\alpha }(t)+{\overset{̅}{w}}_{\alpha }{\overset{̅}{v}}_{\alpha }(t)+{\underline{u}}_{\alpha }^{2}(t)+{\overset{̅}{u}}_{\alpha }^{2}(t\right))dt$ over the set *U*. We now proceed to compute candidates for an optimal solution by applying the Pontryagin's Maximum Principle [24] and begin by defining the Lagrangian to be

(20) $$\begin{array}{cc} & L_{\alpha }\left({\underline{x}}_{\alpha },{\overset{̅}{x}}_{\alpha },{\underline{v}}_{\alpha },{\overset{̅}{v}}_{\alpha },{\underline{z}}_{\alpha },{\overset{̅}{z}}_{\alpha },{\underline{u}}_{\alpha },{\overset{̅}{u}}_{\alpha },\lambda_{1\alpha },\ldots ,\lambda_{6\alpha }\right)\\ & ={\underline{w}}_{\alpha }{\underline{v}}_{\alpha }\left(t\right)+{\overset{̅}{w}}_{\alpha }{\overset{̅}{v}}_{\alpha }\left(t\right)\\ & \;+{\underline{u}}_{\alpha }^{2}+{\overset{̅}{u}}_{\alpha }^{2}+\lambda_{1\alpha }\left({\overset{̅}{\lambda }}_{\alpha }-{\underline{\sigma }}_{\alpha }{\underline{x}}_{\alpha }-{\underline{c}}_{\alpha }{\underline{v}}_{\alpha }\right)\\ & \;+\lambda_{2\alpha }\left({\underline{\lambda }}_{\alpha }-{\overset{̅}{\sigma }}_{\alpha }{\overset{̅}{x}}_{\alpha }-{\overset{̅}{c}}_{\alpha }{\overset{̅}{v}}_{\alpha }\right)+\lambda_{3\alpha }\left(\left({\overset{̅}{k}}_{\alpha }-{\overset{̅}{u}}_{\alpha }\right){\overset{̅}{v}}_{\alpha }-{\underline{a}}_{\alpha }{\underline{z}}_{\alpha }\right)\\ & \;+\lambda_{4\alpha }\left(\left({\underline{k}}_{\alpha }-{\underline{u}}_{\alpha }\right){\underline{v}}_{\alpha }-{\overset{̅}{a}}_{\alpha }{\overset{̅}{z}}_{\alpha }\right)\\ & \;+\lambda_{5\alpha }\left({\overset{̅}{h}}_{\alpha }{\overset{̅}{x}}_{\alpha }-{\underline{\tau }}_{\alpha }{\underline{v}}_{\alpha }\right)+\lambda_{6\alpha }\left({\underline{h}}_{\alpha }{\underline{x}}_{\alpha }-{\overset{̅}{\tau }}_{\alpha }{\overset{̅}{v}}_{\alpha }\right)\\ & \;-\omega_{11}\left({\underline{u}}_{\alpha }-{\underline{u}}_{\alpha }^{min}\right)-\omega_{12}\left({\underline{u}}_{\alpha }^{max}-{\underline{u}}_{\alpha }\right)\\ & \;-\omega_{21}\left({\overset{̅}{u}}_{\alpha }-{\overset{̅}{u}}_{\alpha }^{min}\right)-\omega_{22}\left({\overset{̅}{u}}_{\alpha }^{max}-{\overset{̅}{u}}_{\alpha }\right), \end{array}$$

where *ω*_*ij*_ ≥ 0 are the penalty multipliers satisfying $\omega_{11}({\underline{u}}_{\alpha }^{*}-{\underline{u}}_{\alpha }^{\min})=\omega_{12}({\underline{u}}_{\alpha }^{\max}-{\underline{u}}_{\alpha }^{*})=0$ and $\omega_{21}({\overset{̅}{u}}_{\alpha }^{*}-{\overset{̅}{u}}_{\alpha }^{\min})=\omega_{22}({\overset{̅}{u}}_{\alpha }^{\max}-{\overset{̅}{u}}_{\alpha }^{*})=0$. Thus, the Maximum Principle gives the existence of adjoint variables *λ*_*jα*_, *j* = 1,…, 6, satisfying

(21) $$\begin{array}{c} {\dot{\lambda }}_{1\alpha }=-\frac{\partial L_{\alpha }}{\partial \;{\overset{̅}{x}}_{\alpha }}={\overset{̅}{\sigma }}_{\alpha }\lambda_{2\alpha }-{\overset{̅}{h}}_{\alpha }\lambda_{5\alpha },\\ {\dot{\lambda }}_{2\alpha }=-\frac{\partial L_{\alpha }}{\partial {\underline{x}}_{\alpha }}={\underline{\sigma }}_{\alpha }\lambda_{1\alpha }-{\underline{h}}_{\alpha }\lambda_{6\alpha },\\ {\dot{\lambda }}_{3\alpha }=-\frac{\partial L_{\alpha }}{\partial \;{\overset{̅}{v}}_{\alpha }}=-{\overset{̅}{w}}_{\alpha }+{\overset{̅}{c}}_{\alpha }\lambda_{2\alpha }-\left({\overset{̅}{k}}_{\alpha }-{\overset{̅}{u}}_{\alpha }\right)\lambda_{3\alpha }+{\overset{̅}{\tau }}_{\alpha }\lambda_{6\alpha },\\ {\dot{\lambda }}_{4\alpha }=-\frac{\partial L_{\alpha }}{\partial {\underline{v}}_{\alpha }}=-{\underline{w}}_{\alpha }+{\underline{c}}_{\alpha }\lambda_{1\alpha }-\left({\underline{k}}_{\alpha }-{\underline{u}}_{\alpha }\right)\lambda_{4\alpha }+{\underline{\tau }}_{\alpha }\lambda_{5\alpha },\\ {\dot{\lambda }}_{5\alpha }=-\frac{\partial L_{\alpha }}{\partial {\overset{̅}{z}}_{\alpha }}={\overset{̅}{a}}_{\alpha }\lambda_{4\alpha },\\ {\dot{\lambda }}_{6\alpha }=-\frac{\partial L_{\alpha }}{\partial {\underline{z}}_{\alpha }}={\underline{a}}_{\alpha }\lambda_{3\alpha }, \end{array}$$

where *λ*_*jα*_(*t*_*f*_) = 0, *j* = 1,…, 6, are the transversality conditions. The Lagrangian is minimized with respect to ${\underline{u}}_{\alpha }$ and ${\overset{̅}{u}}_{\alpha }$ at the optimal pair $\left({\underline{u}}_{\alpha }^{*},{\overset{̅}{u}}_{\alpha }^{*}\right)$. So the partial derivatives of the Lagrangian with respect to ${\underline{u}}_{\alpha }$ and ${\overset{̅}{u}}_{\alpha }$ are zero. Since, $\partial L/\partial {\underline{u}}_{\alpha }=2{\underline{u}}_{\alpha }-\lambda_{4\alpha }{\underline{v}}_{\alpha }-\omega_{11}+\omega_{12}=0$, we have ${\underline{u}}_{\alpha }=0.5({\underline{v}}_{\alpha }\lambda_{4\alpha }+\omega_{11}-\omega_{12})$. To determine an explicit expression for the optimal control without *ω*_11_ and *ω*_12_, we consider the following three cases. (i) On the set $\left\{t|{\underline{u}}_{\alpha }^{\min}<{\underline{u}}_{\alpha }^{*}(t)<{\underline{u}}_{\alpha }^{\max}\right\}$, we set *ω*_11_(*t*) = *ω*_12_(*t*) = 0; hence, ${\underline{u}}_{\alpha }^{}=0.5{\underline{v}}_{\alpha }\lambda_{4\alpha }$. (ii) On the set $\left\{t|{\underline{u}}_{\alpha }^{*}(t)={\underline{u}}_{\alpha }^{\max}\right\}$, we set *ω*_11_(*t*) = 0; hence, ${\underline{u}}_{\alpha }^{}={\underline{u}}_{\alpha }^{\max}=0.5\;({\underline{v}}_{\alpha }\lambda_{4\alpha }-\omega_{12})$ which implies that $0.5\;{\underline{v}}_{\alpha }\lambda_{4\alpha }\geq {\underline{u}}_{\alpha }^{\max}$. (iii) On the set $\left\{t|{\underline{u}}_{\alpha }^{*}(t)={\underline{u}}_{\alpha }^{\min}\right\}$, we set *ω*_12_(*t*) = 0; hence, ${\underline{u}}_{\alpha }^{}={\underline{u}}_{\alpha }^{\min}=0.5({\underline{v}}_{\alpha }\lambda_{4\alpha }+\omega_{11})$ which implies that $0.5\;{\underline{v}}_{\alpha }\lambda_{4\alpha }\leq {\underline{u}}_{\alpha }^{\min}$. Combining all the three cases in compact form gives

(22) $${\underline{u}}_{\alpha }^{}\left(t\right)=\max\left({\underline{u}}_{\alpha }^{\min},\min\left(0.5\;{\underline{v}}_{\alpha }\left(t\right)\lambda_{4\alpha }\left(t\right),{\underline{u}}_{\alpha }^{\max}\right)\right).$$

Using similar arguments, we also obtain the following expression for the second optimal control function:

(23) $${\overset{̅}{u}}_{\alpha }^{}\left(t\right)=\max\left({\overset{̅}{u}}_{\alpha }^{\min},\min\left(0.5\;{\overset{̅}{v}}_{\alpha }\left(t\right)\lambda_{3\alpha }\left(t\right),{\overset{̅}{u}}_{\alpha }^{\max}\right)\right).$$

We point out that the optimality system consists of the state system (19) with the initial conditions, the adjoint or costate system (21) with the terminal conditions, together with the expressions (22) and (23) for the control functions. We show the optimal controls and corresponding states and costates satisfying (19) and (21) by a star superscript *∗*. Obviously, if we assume that the set [*λ*_4α_*(*t*), *λ*_3α_*(*t*)]  is a valid **α**-level set of a positive fuzzy number valued function, say ${\tilde{\Lambda }}_{2}^{*}(t)$, then, from (22) and (23),the optimal fuzzy control function ${\tilde{u}}^{*}\in C(T)$ can be written as

(24) $${\tilde{u}}^{*}\left(t\right)=\tilde{\max}\left({\tilde{u}}^{\min},\;\tilde{\min}\left(0.5\;⊗{\tilde{v}}^{*}\left(t\right)⊗{\tilde{\Lambda }}_{2}^{*}\left(t\right),{\tilde{u}}^{\max}\right)\right).$$

Moreover, if we assume that the sets [*λ*_2α_*(*t*), *λ*_1α_*(*t*)]  and [*λ*_6α_*(*t*), *λ*_5α_*(*t*)]  are valid **α**-level sets of fuzzy number valued functions, say ${\tilde{\Lambda }}_{1}^{*}(t)$ and ${\tilde{\Lambda }}_{3}^{*}(t)$, respectively, and the sets $\left[{\dot{\lambda }}_{1\alpha }^{*}(t),{\dot{\lambda }}_{2\alpha }^{*}(t)\right]$, $\left[{\dot{\lambda }}_{3\alpha }^{*}(t),{\dot{\lambda }}_{4\alpha }^{*}(t)\right]$, and $[{\dot{\lambda }}_{5\alpha }^{*}(t),{\dot{\lambda }}_{6\alpha }^{*}(t)]\;$are valid **α**-level sets, then it is easy to see that $(\Theta {\tilde{\Lambda }}_{2}^{*}){⊗}_{Z}\tilde{a}$ and ${\tilde{\Lambda }}_{3}^{*}{⊗}_{Z}\tilde{h}\in F(R)$. Moreover, we can verify that the fuzzy functions ${\tilde{\Lambda }}_{1}^{*}(t)$, ${\tilde{\Lambda }}_{2}^{*}(t)$, ${\tilde{\Lambda }}_{3}^{*}(t)$, and ${\tilde{u}}^{*}$ satisfy the following system of FDEs, with the terminal conditions ${\tilde{\Lambda }}_{j}(t_{f})=(0,0)$, *j* = 1,2, 3, and using the derivative in the second form (ii) (see Definition 9):

(25) $$\begin{array}{cc} & {\dot{\tilde{\Lambda }}}_{1}=\Theta \left\{\left(\Theta {\tilde{\Lambda }}_{1}\right)⊗\tilde{\sigma }⊕{\tilde{\Lambda }}_{3}{⊗}_{Z}\tilde{h}\right\},\\ & {\dot{\tilde{\Lambda }}}_{2}=\Theta \{\tilde{w}⊕\left(\Theta {\tilde{\Lambda }}_{1}\right)⊗\tilde{c}\\ & \;\;\;\;⊕{\tilde{\Lambda }}_{2}⊗\left(\tilde{k}\;\;\Theta_{H}\;\;\tilde{u}\right)⊕\left(\Theta {\tilde{\Lambda }}_{3}\right)⊗\tilde{\tau }\},\\ & {\dot{\tilde{\Lambda }}}_{3}=\Theta \left\{\left(\Theta {\tilde{\Lambda }}_{2}\right){⊗}_{Z}\tilde{a}\right\}. \end{array}$$

Therefore, if we insure that the assumptions mentioned above are satisfied, finding the optimal fuzzy control function can be equivalent to solving a two-point fuzzy boundary value problem (FBVP) which consists of fuzzy system (18) with the initial conditions and the fuzzy system (25) with the final conditions, together with the expression $\tilde{u}(t)=\tilde{\max}({\tilde{u}}_{}^{\min},\tilde{\min}(0.5⊗\tilde{v}(t)⊗{\tilde{\Lambda }}_{2}(t),{\tilde{u}}_{}^{\max}))$ for the control function. Here, we verify that ${\tilde{\Lambda }}_{1}^{*}(t)$, ${\tilde{\Lambda }}_{2}^{*}(t)$, ${\tilde{\Lambda }}_{3}^{*}(t)$, and ${\tilde{u}}^{*}$ satisfy the second equation in (25) and the rest can be verified similarly. Since the functions *λ*_3α_* and *λ*_4α_* are positive from the two last equations in (21), we conclude that *λ*_5α_* and *λ*_6α_* are negative functions. Moreover, from the two first equations in (21), we have

(26) $$\begin{array}{cc} & \lambda_{1\alpha }^{*}\left(t\right)=\overset{t_{f}}{\underset{t}{\int }}\{{\overset{̅}{h}}_{\alpha }\cosh\left\{\sqrt{{\overset{̅}{\sigma }}_{\alpha }{\underline{\sigma }}_{\alpha }}\left(\tau -t\right)\right\}\lambda_{5\alpha }^{*}\left(\tau \right)\\ & \;\;\;\;\;\;+\sqrt{\frac{{\overset{̅}{\sigma }}_{\alpha }}{{\underline{\sigma }}_{\alpha }}}{\underline{h}}_{\alpha }\sinh\left\{\sqrt{{\overset{̅}{\sigma }}_{\alpha }{\underline{\sigma }}_{\alpha }}\left(\tau -t\right)\right\}\lambda_{6\alpha }^{*}\left(\tau \right)\}d\tau ,\\ & \lambda_{2\alpha }^{*}\left(t\right)=\overset{t_{f}}{\underset{t}{\int }}\{\sqrt{\frac{{\underline{\sigma }}_{\alpha }}{{\overset{̅}{\sigma }}_{\alpha }}}{\overset{̅}{h}}_{\alpha }\sinh\left\{\sqrt{{\overset{̅}{\sigma }}_{\alpha }{\underline{\sigma }}_{\alpha }}\left(\tau -t\right)\right\}\lambda_{5\alpha }^{*}\left(\tau \right)\\ & \;\;\;\;\;\;+\;{\underline{h}}_{\alpha }\cosh\left\{\sqrt{{\overset{̅}{\sigma }}_{\alpha }{\underline{\sigma }}_{\alpha }}\left(\tau -t\right)\right\}\lambda_{6\alpha }^{*}\left(\tau \right)\;\}d\tau , \end{array}$$

which implies that the functions *λ*_1α_* and *λ*_2α_* are negative; hence,

(27) $$\begin{array}{cc} & {\underline{\Theta \;\left\{\tilde{w}⊕\left(\Theta {\tilde{\Lambda }}_{1}^{*}\right)⊗\;\tilde{c}⊕{\tilde{\Lambda }}_{2}^{*}⊗\left(\tilde{k}\Theta_{H}{\tilde{u}}^{*}\right)⊕\left(\Theta {\tilde{\Lambda }}_{3}^{*}\right)⊗\tilde{\tau }\right\}}}_{\alpha }\\ & =-{\bar{\left\{\tilde{w}⊕\left(\Theta {\tilde{\Lambda }}_{1}^{*}\right)⊗\;\tilde{c}⊕{\tilde{\Lambda }}_{2}^{*}⊗\left(\tilde{k}\Theta_{H}{\tilde{u}}^{*}\right)⊕\left(\Theta {\tilde{\Lambda }}_{3}^{*}\right)⊗\tilde{\tau }\right\}}}_{\alpha }\\ & =-\;\left\{{\overset{̅}{w}}_{\alpha }-\lambda_{2\alpha }^{*}{\overset{̅}{c}}_{\alpha }+\lambda_{3\alpha }^{*}\left({\overset{̅}{k}}_{\alpha }-{\overset{̅}{u}}_{\alpha }^{*}\right)-\lambda_{6\alpha }^{*}{\overset{̅}{\tau }}_{\alpha }\right\}={\dot{\lambda }}_{3\alpha }^{*}. \end{array}$$

We summarize our results in the following theorem.

**Theorem 13**

Assume that ${\tilde{\Lambda }}_{2}^{*}(t)$ is a positive fuzzy function on T. A candidate for fuzzy optimal control is given by (24), if the fuzzy functions ${\tilde{u}}^{*}(t)$, ${\tilde{x}}^{*}(t)$, ${\tilde{z}}^{*}(t)$, and ${\tilde{v}}^{*}(t)$ satisfy (18) using the derivative in the first form (i) and ${\tilde{u}}^{*}(t)$, ${\tilde{\Lambda }}_{1}^{*}(t)$, ${\tilde{\Lambda }}_{3}^{*}(t)$, and ${\tilde{\Lambda }}_{2}^{*}(t)$ satisfy (25) using the derivative in the second form (ii).

**Figure 11:**
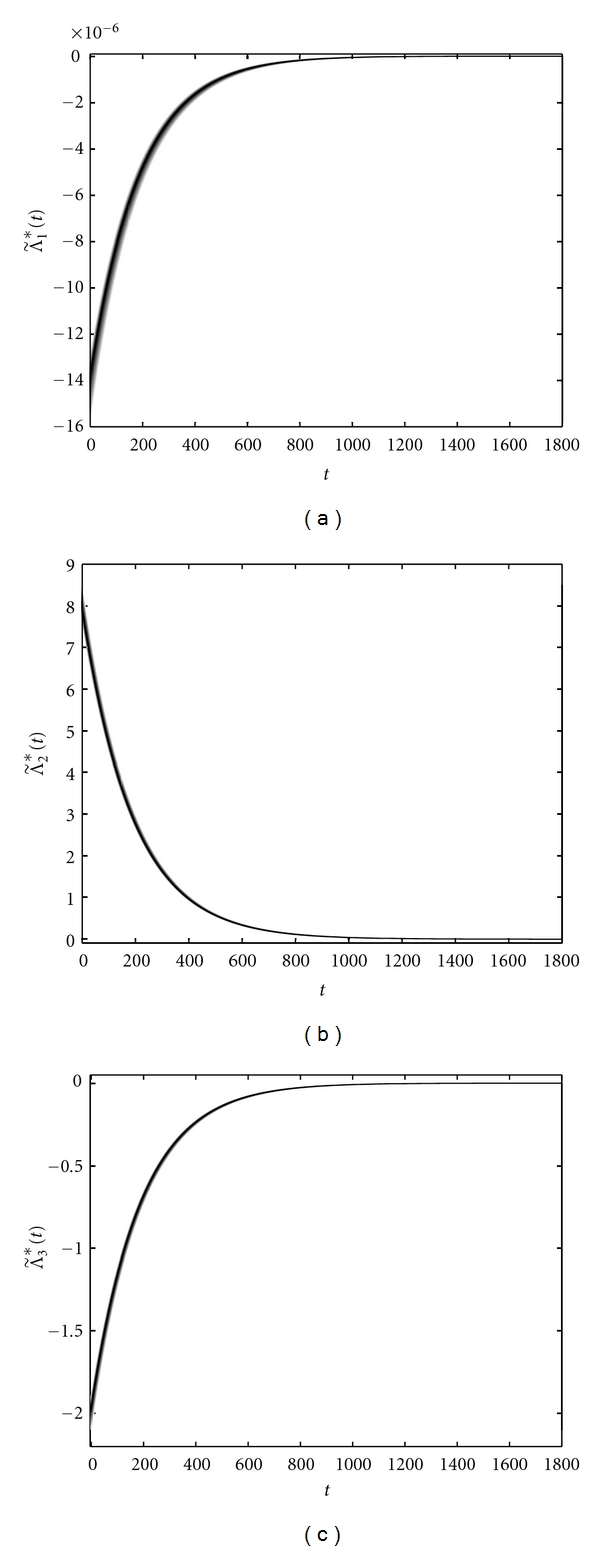
Optimal fuzzy adjoint variables ${\tilde{\Lambda }}_{1}^{*}$(a), ${\tilde{\Lambda }}_{2}^{*}$(b), and ${\tilde{\Lambda }}_{3}^{*}$(c) versus time.

**Figure 12:**
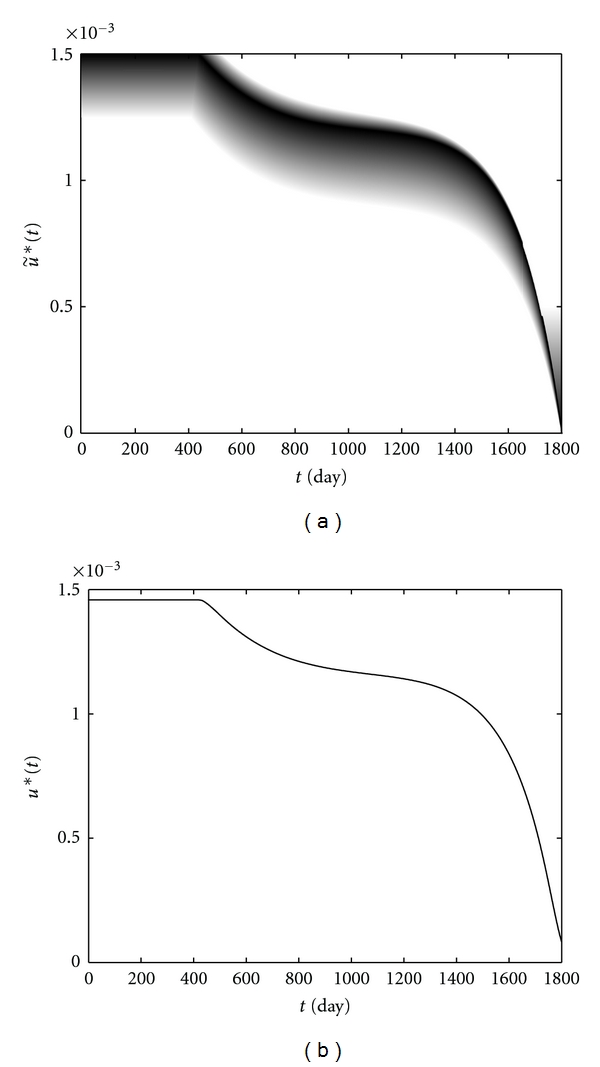
Optimal fuzzy control (a) and its WCOG (b) versus time.

**Figure 13:**
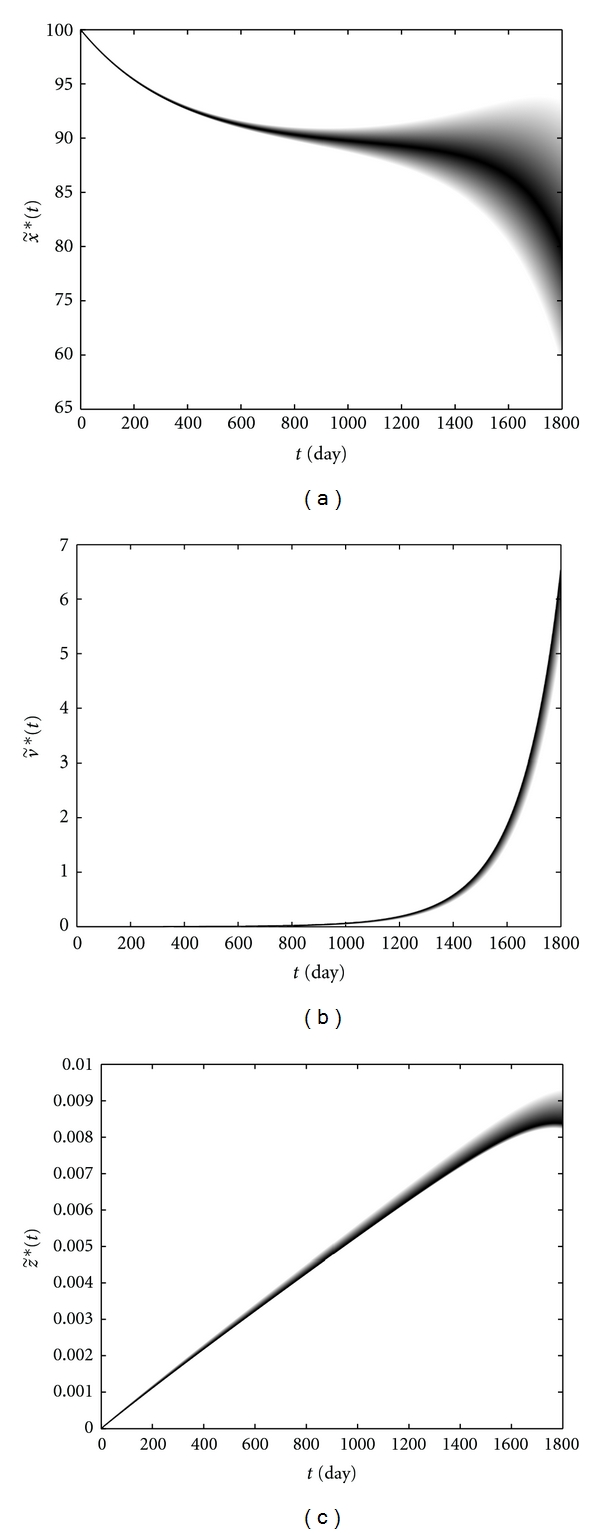
CD4+ T-cells level (a), the viral load (b), and CTLs level (c) in presence of treatment versus time in patient *W*.

**Figure 14:**
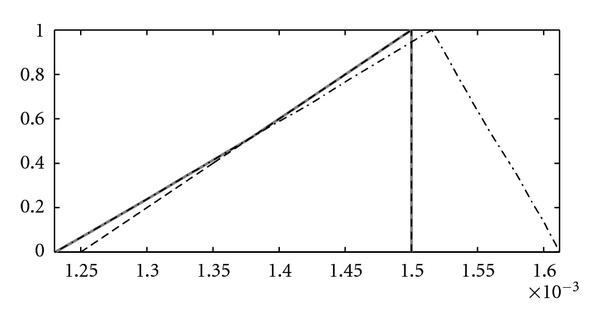
${\tilde{u}}_{}^{\max}$(- -), $0.5\;⊗{\tilde{v}}^{*}(425)⊗{\tilde{\Lambda }}_{2}^{*}(425)$ (–·–), ${\tilde{u}}^{*}(425)=\tilde{\min}(0.5\;⊗{\tilde{v}}^{*}(425)⊗{\tilde{\Lambda }}_{2}^{*}(425),{\tilde{u}}_{}^{\max})$ (—).

**Figure 15:**
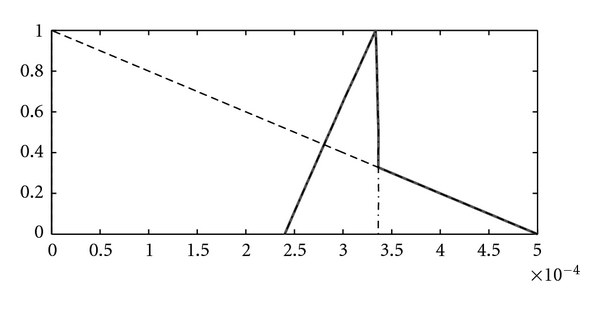
${\tilde{u}}_{}^{\min}$ (- -), $0.5⊗{\tilde{v}}^{*}(1750)⊗{\tilde{\Lambda }}_{2}^{*}(1750)$ (–·–), ${\tilde{u}}^{*}(1750)=\tilde{\max}(0.5⊗{\tilde{v}}^{*}(1750)⊗{\tilde{\Lambda }}_{2}^{*}(1750),{\tilde{u}}^{\min})$ (—).

We have solved the optimality system corresponding to patient *W* with the fuzzy weight $\tilde{w}=(0.24\times 10^{-5},0.24\times 10^{-6})$ and the fuzzy bounds ${\tilde{u}}^{\min}=(0,0,0.5\times 10^{-\;3})$ and ${\tilde{u}}^{\max}=(0.15\;\times \;10^{-2},0.25\;\times \;10^{-\;3},0)$, during the time interval [0, 1800] by using the gradient method [24]. Numerical results show that the optimal adjoint variables satisfy the assumptions mentioned above.  Figure 11 shows the optimal fuzzy adjoint variables, while the optimal fuzzy control function ${\tilde{u}}^{*}$ and its WCOG as a real-valued output u* indicating an optimal drug regimen are depicted in Figure 12. Moreover, the optimal fuzzy states ${\tilde{x}}^{*}(t)$, ${\tilde{z}}^{*}(t)$, and ${\tilde{v}}^{*}(t)$ indicating the immune cells level and the HIV viral load in presence of treatment are shown in Figure 13. The values of ${\tilde{u}}^{*}$ on the 425th and the 1750th days are, respectively, shown in Figures 14 and 15, for the sake of clarity. 

 The proposed treatment by Figure 12, which its intensity is decreasing during the time interval [0, 1800], reduces the proliferation rate of viruses considerably. Therefore, the HIV viral load is very low as shown in Figure 13(b). A very low viral load slows the destruction of CD4+ T-cells which are due to contact with virus particles; hence, a CD4+ T-cells level below 70% is not possible in a treated patient as shown in Figure 13(a). From Figure 13(c), a high level of CD4+ T-cells and a low viral load lead to establishment of a lasting CTL-mediated immune response.

## 5. Conclusion

In this paper, we proposed a fuzzy mathematical model of HIV dynamic. Simulation results show that the proposed three-dimensional FDEs can describe the ambiguous immune cells level and the HIV viral load which are due to existing patients with various strength of their immune system. Moreover, we utilized the proposed fuzzy model and studied a fuzzy optimal control problem minimizing both the viral load and drug costs. Using the Pontryagin's Maximum Principle leads us to the conclusion that the fuzzy optimal control function may not exist in general, but an optimality system containing fuzzy state and fuzzy adjoin equations is derived under certain assumptions. Motivated by these results, we tend to exploit necessary and sufficient conditions to the existence of fuzzy solutions for linear fuzzy optimal control problems. We expect to address these problems in further works.
